# A novel graph mining approach to predict and evaluate food-drug interactions

**DOI:** 10.1038/s41598-022-05132-y

**Published:** 2022-01-20

**Authors:** Md. Mostafizur Rahman, Srinivas Mukund Vadrev, Arturo Magana-Mora, Jacob Levman, Othman Soufan

**Affiliations:** 1 Department of Computer Science, St. Francis Xavier University, Antigonish, NS Canada; 2 Drilling Technology Team, EXPEC Advanced Research Center, Saudi Aramco, Dhahran, 31311 Saudi Arabia

**Keywords:** Computer science, Data processing

## Abstract

Food-drug interactions (FDIs) arise when nutritional dietary consumption regulates biochemical mechanisms involved in drug metabolism. This study proposes FDMine, a novel systematic framework that models the FDI problem as a homogenous graph. Our dataset consists of 788 unique approved small molecule drugs with metabolism-related drug-drug interactions and 320 unique food items, composed of 563 unique compounds. The potential number of interactions is 87,192 and 92,143 for disjoint and joint versions of the graph. We defined several similarity subnetworks comprising food-drug similarity, drug-drug similarity, and food-food similarity networks. A unique part of the graph involves encoding the food composition as a set of nodes and calculating a content contribution score. To predict new FDIs, we considered several link prediction algorithms and various performance metrics, including the precision@top (top 1%, 2%, and 5%) of the newly predicted links. The shortest path-based method has achieved a precision of 84%, 60% and 40% for the top 1%, 2% and 5% of FDIs identified, respectively. We validated the top FDIs predicted using FDMine to demonstrate its applicability, and we relate therapeutic anti-inflammatory effects of food items informed by FDIs. FDMine is publicly available to support clinicians and researchers.

## Introduction

Drugs bind to targeted receptors on the surface of cells or enzymes to regulate the rate of chemical reactions. These chemical reactions may be relied upon to treat different diseases and considerably enhance the patients’ prognoses. However, drug overdoses or drug interactions may cause critical adverse health conditions. Although the impact of the drugs depends on the affinity of the drug to bind to a specific cell/enzyme receptor, its effectiveness also depends on other factors, such as when taken alongside other food or drugs. Ideally, drug effects should be consistent across all patients for whom food ingredients (or other medical products) do not affect drug response^1^. However, several studies^2,3^ have demonstrated the impact of certain foods, decreasing or increasing the activity of different drugs (food-drug interactions—FDI).

 Drug plasma concentrations are frequently affected by FDIs, which may significantly increase or decrease the effectiveness of the drug^4^. These alterations can occur in three ways: they can boost the actions of drugs (i.e., increased metabolism), reduce the actions of drugs (i.e., decreased bioavailability), or create an adverse effect.

FDIs can be classified into two primary mechanisms: pharmacokinetic (PK) interactions and pharmacodynamic (PD) interactions^5^. PK interactions denote the circumstance when foods alter processes related to absorption, metabolism, distribution, and excretion of medications. For example, for a brief period of time after consumption, grapefruit juice slows the metabolism of cyclosporine (e.g.: cytochrome P450 enzymes)^6,7^. PD interactions are caused by specific interactions between a drug and a food component, resulting in a pharmacological effect^8^. An example PD interaction is found in a diet high in vitamin K that antagonizes the therapeutic effects of warfarin (which is used for blood clot treatments)^5^.

FDIs can affect drug absorption levels and, therefore, are known to impact drug discovery^9^. For example, *Moringa oleifera* leaf extract has been used to inhibit cancer cells and to increase the efficacy of chemotherapy in humans^10–12^. The roots of Erythroxylum pervillei provide pervilleines A, B, C, and F, effective inhibitors of P-glycoprotein, which is linked to multidrug resistance and low cancer therapeutic response^13^. These are only a few examples that demonstrate the importance of understanding the interactions of food constituents and dietary supplements (containing different chemicals and phytochemicals) with drugs. Consequently, understanding FDIs has the potential to assist physicians, researchers, and patients in reducing adverse drug events (ADEs).

Earlier research has been mostly based on clinical studies or literature reviews that focus on specific drug interactions with a limited set of foods^5,8,14,15^. Based on analyzing PD or PK alterations, these studies examine how food items can affect the efficacy of specific drugs. Some studies have examined FDI interactions with the types and number of drugs used (e.g., drugs used for chemotherapy, drugs used as anticoagulants)^16–18^. Although these studies provided valuable information to physicians about the potential importance of FDIs, the level of novel exploration analytic techniques is limited in the literature. Thus computational approaches have the potential to predict/discover novel FDIs, which may inform a higher standard of patient care.

### Related work

Cheminformatics studies have achieved remarkable results in drug-drug interactions (DDIs), drug-target interactions (DTIs), and new drug discovery. Multiple computational models have been developed for detecting how a particular drug pair interacts towards the discovery of new drugs. The adoption of different machine learning models is rapidly increasing in drug discovery^19^. These models have been used for the discovery of novel DDIs. For instance, a deep learning model was implemented to predict the pharmacological effects of DDIs using the structural similarity profile (SSP), target gene similarity profiles, and gene ontology (GO) term similarity profiles of known drug pairs^20^. Recently, DeepDDI was developed as a multi-label classification model that calculates structural similarity profiles (SSP) of DDIs^21^. DeepDDI employs principal components analysis to reduce the feature set size before feeding the rotated data into a feed-forward deep neural network (DNN)^21^. Another predictive model was trained to distinguish unknown biological effects of inactive ingredients that are recognized as safe compounds in food^22^. A general-purpose method, named Alternative Drug-Drug Interaction, was developed for DDI predictions^23^. Three combined methods were used, including text mining, deep learning, and graph clustering. Feng et al. proposed DPDDI to predict DDIs without considering biological and chemical properties^24^. The authors used graph convolution networks (GCN) and DNN for prediction. GCN explores low-dimensional feature representations of drugs by identifying the topological association of drugs in the DDI network.

Several chemoinformatics studies have successfully demonstrated the application of computational models DTI prediction. Yo et al.^25^ trained a deep learning model to predict DTIs using a network representation. The solution is a linear classification model that uses the least absolute shrinkage and selection operator (LASSO) and LASSO-DNN, which assisted in feature extraction towards successful DTI predictions. In a previous study, we developed DASPfind (drug activity shortest-paths finding)^26^, a novel computational method to infer novel drug-target interactions (or DTIs), where the target is a protein, from a graph structure using a simple path query of length two or three. The graph included similarities of protein–protein, drug-drug, and known drug-protein interactions. A random forest model was trained on extracted features that summarize information of the graph built from known DTIs and multiple similarities among the drug-drug and target-target interactions^27^. A variety of studies have developed link prediction approaches to predict DTIs. Lu et al.^28^ used link prediction based on similarity indices for DTI prediction. Fokoue et al.^29^ developed the Tiresias framework that uses large-scale similarity-based link prediction based on different drug data to assess DDIs. The framework uses a large-scale logistic regression model for DDI prediction.

Although interaction identification technologies have made significant advances in these chemoinformatics models for DDIs and DTIs, FDIs remain poorly addressed. This is primarily due to a lack of FDI resources, as it is usually challenging to extract a significant number of curated interactions. In addition, for FDI, there is no gold standard dataset available for evaluation at this time. Recently, FooDB^30,31^ was developed as a well-structured and annotated database providing food items and compound composition. Although there is no gold standard dataset for evaluation as in DTIs, we propose using known DDIs. Given the homogenous nature of our graph representations (i.e., all nodes are chemicals), we can resort to subnetworks for evaluation. To the best of our knowledge, this is the first study on developing a homogenous graph mining framework for food-drug interactions. A table that summarizes related work is provided in Supplementary file 1: Table S18.

This study proposes FDMine, a framework that analyzes FooDB^30,31^ and DrugBank^32^ databases to create a comprehensive dataset of small molecules with known food-food interactions (FFIs), DDIs, and FDIs. FDMine uses the simplified molecular-input-line-entry system (SMILE) description to establish similarity profiles and link prediction algorithms to predict the FDIs. The proposed framework uses two different kinds of representations (disjoint and joint) graphs consisting of three connected subnetworks. These subnetworks are drug-drug similarity, food-drug similarity, and food-food similarity. The rationale behind this approach is to capitalize on the similarity information of different subnetworks and combine it with building a homogenous graph. We consider a unique representation of food items, their compound composition, and the contribution of each compound. After building the graph network, the framework implements a comprehensive set of different link prediction algorithms to predict potential FDIs. The shortest path-based method has achieved a precision 84%, 60% and 40% for the top 1%, 2% and 5%, respectively. In the joint version of the graph, FDMine recovered 27,448 links on average from 27,612 available (i.e., 99.4% recovery with a standard deviation of 5.1e^−4).

This paper is organized as follows: the Methods section contains a detailed description of the dataset prepared and generated from DrugBank and FooDB, followed by an illustration of the graph representation, structure similarity profile, sparse matrix representation, and the procedure to update the similarity score. In addition, an explanation is provided for the evaluation metrics used to evaluate the performance of FDMine, including the train and test dataset, ground truth dataset, and different evaluation procedures. The description of the FDMine framework is provided in the Methods. The Results and Discussion section contains the reported performance scores of FDMine based on the different datasets, comparisons, and evaluation criteria. Literature validation for the top predictions produced by FDMine are provided, including a discussion on the limitations of the proposed framework. The Conclusion section summarizes the main results and provides a highlight of future work.

## Methods

### Databases and datasets preparation

#### DrugBank

We used the DrugBank (v 5.1.7) database, which contains substantial drug target information (i.e., sequence, pathway, and structure) as well as specific information for each drug (i.e., chemical, medicinal, and pharmacological data)^32–34^. A total of 13,680 distinct drugs are represented in the database. Approved, experimental, investigational, nutraceutical, and withdrawn are the five categories of drugs in DrugBank. Drugs can be differentiated as small molecules or biotechnology-driven. The database provides access to the SMILE strings of the drug molecules and reports drug-drug interactions^33^.

In this study, we considered the drugs assigned to the approved drug group and have small molecules. This resulted in 1683 drugs. We further reduced this set of molecules by considering only those having “metabolism (increase or decrease)” related interactions, resulting in 788 unique approved small molecule drugs. FDI interactions are mainly detected with metabolic mechanisms^14^. The details of the drug extraction procedure from the DrugBank dataset can be found in Supplementary file 1: Fig. S1.

#### FooDB

We used the JSON formatted^30,31^ FooDB version 1.0 dataset, which includes numerous records on foods, compounds, content, nutrients, and health effects. We used the FooDB content dataset, which directly matched foods to food compound composition. Then, we cleaned the retrieved data by discarding the list of unknown data elements using the criteria “DATABASE" as the “citation type” and “COMPOUND” as “source type”. This provides a more accurate source of information. Finally, we only considered the food items mapped to a compound, resulting in 16,230 objects for further analysis.

After the parsing step, we mapped the resulting dataset with the “Compound” information to collect the required details for each compound, including the SMILE description and the content contribution. In FoodDB, the content range of each compound within a food item is presented (e.g., Strawberry has a content range of Potassium of 0.000–187.000 mg/100 g). Finally, we have the SMILE description of the corresponding compounds along with the contribution of each food compound.

We filtered data from the health effects dataset to find food compounds with reported health impacts on the human body. The resulting dataset contains 8846 objects, including 320 unique foods, and 563 unique food compounds having 179 unique health effects. Benzoic acid from American cranberry having an allergenic health effect is one example food compound with reported health impacts on the human body.

Since the same compounds can be found in different foods, it is necessary to store these data with a naming convention that allows us to correctly differentiate each food with its composition. In this study, we used the following naming convention: *FOODXXXX_FDBXXXXX_CompoundName*. For example, the data entries “*FOOD00005_ FDB000633_ Kaempferol*” and “*FOOD00008_ FDB000633_ Kaempferol*” refer to the same compound Kaempferol with the compound identifier *FDB000633* from two different foods (*FOOD00005* and *FOOD00008*). Each compound can be treated differently based on the reported content range in the food item.

A diagram illustrating the data preparation steps from FooDB database can be found in Supplementary file 1: Fig. S2. DrugBank (v 5.1.7) database and FooDB version 1.0 dataset are publicly available. All the methods were performed in accordance with the relevant guidelines and regulations.

#### Food composition and compound contribution

Each food item is composed of a set of chemical compounds. Clearly, the “amount of the original content” of any compound is not the same for each food. For example, the amount of the phytic acid in carrot is 5270.000 ml/100 g, and buckwheat is 1800.000 ml/100 g. Carrot contains approximately three times more phytic acid than buckwheat by mass. Therefore, the contribution of the phytic acid is different for carrot and buckwheat. Consequently, we used the following equation to calculate the contribution of each compound for each food based on the amount contained in the food:

1 $$ Contribution\; score\;\left( {normalized} \right) = \frac{Compound\; original \;content\; \in \,a\; food\; item}{{Total \;original\; content \;of\; all \;compounds\; \in \;a \;food}} $$

The range of the normalized contribution is from 0 to 1. Where a 0 and 1 contribution refer to a food compound with no contribution or full contribution, respectively. Assuming the amount of a compound in a food is given in mg/100 g and all compounds that a food is comprised of are considered, the original content of all compounds should always be 100 g/100 g = 1. Yet, some of the compounds might be excluded if some relevant information is missing from FoodDB (e.g., missing content value or missing health effect information or missing reference database). Hence, normalization is necessary for computing the contribution score.

In the graph, the food item and its compound composition are represented as separate nodes. The normalized contribution score scales edge weights of links connecting compounds to the food item. More details and an example on the contribution score of food compounds is provided in Supplementary file 1: Table S1.

### Homogenous graph representation

We consider a set of food compounds, $F = \left\{ {f_{1} ,f_{2} , \ldots ,f_{m} } \right\}$ and a set of drugs, $D = \left\{ {d_{1} ,d_{2} , \ldots ,d_{n} } \right\}$ with $m$ representing the number of food compounds and *n* representing the number of drugs included. We merged all drugs and food compounds into a single graph. So, in our representation, we have a set of drug and food compounds $FD = \left\{ {f_{1} ,f_{2} , \ldots ,f_{m} ,d_{1} ,d_{2} , \ldots ,d_{n} } \right\}$. Then, we considered the set of $m*n$ dimensional structure similarity matrices between drugs, food compounds, and food-drug pairings. The strength of similarity is measured by a score between [0, 1]. A similarity score close to 0 implies that two items are not identical to each other, where the most similar items are represented by a similarity score close to 1. Using this similarity assessment, we derived a homogenous graph. For this homogenous graph, we will apply different path categories and neighborhood-based similarity-based algorithms to predict the novel FDIs.

### Structure similarity profile

A structural similarity profile (SSP) is a feature vector containing a unique numerical representation after acquiring structural features of individual food compounds and drugs. The SSP contains pairwise structural similarity scores obtained from the comparison among all of the 788 approved small molecule drugs of DrugBank and 8846 unique food compounds. The Tanimoto coefficient measured structural similarity between a pair of nodes (*i.e., drug-drug, food-food, and food-drug*). This coefficient is an efficient way to calculate structure similarity based on the chemical fingerprint^35,36^. The Tanimoto coefficient is defined as the number of common chemical fingerprints compared to all chemical fingerprints of the two drugs. Chemical fingerprints for each drug were calculated using Morgan/Circular fingerprints^37^ (also known as extended-connectivity fingerprint ECFP4^38^) that is widely used. ECFP4 demonstrated the best performing fingerprints in the target prediction benchmarks^39,40^ and in small molecule virtual screening^41^. The calculating procedure of the SSP can be found in Supplementary file 1: Fig S3.

### Sparse matrix representation

We used the similarity profile to derive a sparse matrix representation, which is used for plotting the graphs. In this matrix, we eliminated the zero entries and applied a threshold since some similarity scores contain trivial values and thus may not be indicative of significant changes. For determining the threshold, we have considered the similarity score distribution. Most similarity values lie between 0.3 and 0.6, hence, selecting a high similarity value may drastically change the dataset size. Also, a high threshold will always lead to potential pairs having increased probability of interaction. Several studies have referred to different values in the range of 0.5–0.85 for applying a similarity threshold for the Tanimoto coefficient^42–44^. While a higher threshold reduces the probability of reporting spurious findings, it can limit the number of genuinely novel predictions. Table 1 highlights the number of links of each subnetwork after applying a range of similarity thresholds. Compared to a threshold of 0.6, a value of 0.7 would result in 75% fewer possible FDIs, thus, we selected 0.6 at this step. It should be noted that this parameter is provided as an input argument for the user of FDMine.

**Table 1:** Number of links in the graph after applying different Tanimoto similarity thresholds.

Tanimoto threshold	Total links	DD links	FF links	FD links
> = 0.5	5,392,354	14,298	5,228,607	149,449
> = 0.6	4,177,383	2926	4,167,202	7255
> = 0.7	3,834,135	920	3,831,336	1879

### Updating similarity scores using food-compound contribution

We obtained a total of 4,177,383 similarities using the SSP. Then, we multiplied the similarity score by the normalized contribution of the food compound (Eq. 1). As illustrated in Supplementary file 1: Table S2, when we have a food-drug pair (see row 1), we multiply the similarity score by the contribution of the food compound. Similarly, we multiplied the similarity score by the higher contribution of the food compound. For example, the contribution of the *FOOD00006_ FDB000474_ L-Lysine* is 0.007301117, and the *FOOD00006_ FDB000556_ L-Alanine* is 0.009780473. So, we have considered the maximum value of 0.009780473 to update the similarity score. For drug pairs, similarity scores were preserved.

2 $$ Score = Prior \;Score\left( {SSP} \right)*Contribution \;of\; Food\; Compound $$

After updating the similarity scores in the graph, we consider another threshold using the contribution score. Here, we consider a more relaxed range of thresholds (0.3, 0.4, 0.5 and 0.6) applied to the Tanimoto coefficient. In our literature validation, we prepare and discuss another batch of results using a similarity score of 0.3, though a value of 0.5 has been employed to generate our primary findings. For a threshold of 0.5, we ended up with 87,192 interactions and 92,143 for the disjoint and joint datasets respectively. In Supplementary file 1: Table 3lists the number of interactions for the considered range.

### Link prediction algorithms

After applying the similarity thresholds, the generated graph had several disjoint subgraphs. We call this the disjoint version. Some link prediction algorithms like Adamic and Adar Coefficient (AA), as well as Common Neighbour (CN), cannot handle the disjoint version, therefore, we prepared a joint graph. We chose any node (randomly) from each subgraph and added an edge to link all subgraphs to make the joint graph network. Then, a very small edge weight of 1e-5 was assigned to the newly added links (see Table S22 for results using an edge weight of 1), limiting their effect on generating biased hypotheses. We generated results for both versions. A detailed description is available in Supplementary file 1: Fig S4.

#### Path category-based algorithm

Our goal is to use the created homogenous graph to predict novel (i.e., unknown) FDIs. A homogenous graph is one where all nodes are of the same type. Nodes in our graph are chemicals, which discerns this approach from DTI heterogenous graphs (e.g., drug-protein). One class of algorithms runs the shortest path to find candidate interactions for the considered food and drug pair. Here, we employed 2-length and 3-length pathways. For example, a 2-length path is “*Drug1-Food1-Food2*” (see Supplementary file 1: Fig S5) connects the Drug1 node with the Food2 node through the similarity between “*Drug1 and Food1*” and “*Food1 and Food2*”. This is defined as a D-F-F path. As illustrated in Supplementary file 1: Fig S5, the gold color circle denotes the food node and silver color circle denotes the drug node. There are 8 possible combinations of paths (i.e., *Drug-Drug-Drug, Drug-Food-Drug, Food-Food-Food, Food-Drug-Food, Drug-Drug-Food, Drug-Food-Food, Food-Drug-Food, and Food-Food-Food*).

Any path can be taken to predict novel interactions. The same applies for 3-length pathway prediction. For example, we can get another new link using 3-path length (*Food-Food-Drug-Food*). The score for the newly predicted link is calculated according to Eq. (3), where $P$ is the path, $n$ is the total number of paths, $w$ is the weight of the path and $P_{w}$ is the path weight:

3 $$ score = Min\mathop \sum \limits_{p = 1}^{n} P_{w} $$

Dijkstra's algorithm was used for finding the shortest path between all pairs of nodes in the graph. For each pair of FDI, the Dijkstra algorithm will find the path with the smallest values (i.e., lower similarity scores). In order to compensate for this issue, we rank the final list in *descending* order, giving preference for interactions with higher similarity. For example, given Dijkstra score(DrugA-FoodX) = 1.12 and Dijkstra score(DrugB-FoodY) = 2, the preference will be assigned to DrugB-FoodY. Another approach to handle the challenge of Dijkstra’s algorithm reporting the shortest path, would have been to invert the similarity scores (so that they represent dissimilarities) and then use a standard ascending rank. ​After applying Dijkstra’s algorithm, we performed filtering with a path length of 2 or 3, and considered the results as possible interactions.

#### Neighbourhood-based similarity-based link prediction

In the link prediction, given a graph $G$, the main aim is to predict new edges (drug-food) from the existing graph. Predictions are useful to suggest unknown relations (or interactions) based on edges in the observed graph. In the link prediction, we build a similarity measure between pairs of nodes and link the most similar nodes. Link prediction algorithms are widespread in many application domains such as, identifying protein–protein interactions^45^, drug-drug interactions^29^, DTIs^28^, social networks^46^, reconstructing networks^47^, document recommendation, recommendation systems^48^, biological networks^49^, disease prediction^50^, bipartite networks^51^, etc.

Here, we applied six different types of link prediction algorithms. They are, Adamic and Adar Coefficient (AA)^50,52^, Common Neighbor (CN)^28,50,53^, Jaccard Coefficient (JAC)^28,50^, Resource Allocation (RA)^50,54,55^, Multiple Paths of Length L = 3 (L3)^45,56^, and Dice Coefficient (Dice). All of these algorithms have respective scoring functions. Each of these algorithms assigns a score for the new predicted links.

#### Adamic and adar coefficient (AA)

The Adamic and Adar Coefficient (AA) gives preference to node pairs with more common neighbors but with a lower degree. If there are no common neighbors for a node pair, then the AA score is 0. The AA measure is formulated to connect node pairs that have common neighbors.

4 $$ S_{AA} \left( {a,b} \right) = \mathop \sum \limits_{z \in \Gamma \left( a \right) \cap \Gamma \left( b \right)} \frac{1}{{logk_{z} }} $$

Here, $a$ and $b$ are two nodes, and $z$ denotes a common neighbor to both $a$ and $b$. $k$ is the degree of node $z$.

#### Common neighbor (CN)

In the Common Neighbor (CN) algorithm, the score for link prediction is computed by finding the number of common neighbors between two distinct nodes. Where, $a$ and $b$ are two nodes. Γ($a$) and Γ($b$) denote the set of neighbors of nodes $a$ and $b$, respectively.

5 $$ S_{CN} \left( {a,b} \right) = \left| {\Gamma \left( a \right) \cap \Gamma \left( b \right)} \right| $$

#### Jaccard coefficient (JAC)

The JAC measure considers only node pairs that have at least one common neighbor. The JAC measure gives equal weight to all common neighbors and does not consider the degree of the common neighbors. The JAC measure gives preferences to node pairs that share a larger fraction of their neighbor. The JAC measure always ranges from 0 to 1 irrespective of the size of the neighborhoods of the vertices. The formula is given below to calculate the JAC. Γ($a$) and Γ($b$) denote the set of neighbors of nodes $a$ and $b$, respectively.

6 $$ S_{Jaccard} \left( {a,b} \right) = \frac{{\left| {\Gamma \left( a \right) \cap \Gamma \left( b \right)} \right|}}{{\left| {\Gamma \left( a \right) \cup \Gamma \left( b \right)} \right|}} $$

#### Resource allocation (RA)

Resource Allocation (RA) calculates the score based on irregular nodes connecting node $a$ and $b$. The number of resources node $a$ receives from node $b$ through indirect links is called the similarity index. In the RA, each intermediate link contributes a unit of the resource. The RA is also symmetric. $z$ denotes a common neighbor of both $a$ and $b$ nodes and k denotes the degree of node $z$.

7 $$ S_{RAI} \left( {a,b} \right) = \mathop \sum \limits_{z \in \Gamma \left( a \right) \cap \Gamma \left( b \right)} \frac{1}{{k_{z} }} $$

#### *Multiple paths of length L* = *3 (L3)*

Links of high degree nodes prompt multiple and unspecific shortcuts in the network, resulting in biased predictions. This can be avoided by using a proper degree of normalization. Such a degree of normalization is significant for *L3*. To eliminate potential degree biases caused by lower degree nodes, we assign a degree normalized *L3* score to each node pair $a$ and $b$. Here, *u* and *v* are intermediate nodes in the 3-length path, $A_{au}$, $A_{uv}$, $A_{vb}$ are the link weight, $K_{u}$, $K_{v}$ are the degree of node *u* and *v* respectively.

8 $$ L3_{ab} = \mathop \sum \limits_{u,v \in L3} \frac{{A_{au} A_{uv} A_{vb} }}{{\sqrt {k_{u} k_{v} } }} $$

#### Dice coefficient

Dice coefficient is similar to the Jaccard Coefficient (JAC). The Dice coefficient is calculated using Eq. (9), where, $a$ and $b$ are two nodes and Γ($a$) and Γ($b$) denote the set of neighbors of nodes $a$ and $b$, respectively.

9 $$ S_{Dice} \left( {a,b} \right) = \frac{{2*\left| {\Gamma \left( a \right) \cap \Gamma \left( b \right)} \right|}}{{\left| {\Gamma \left( a \right) \cup \Gamma \left( b \right)} \right|}} $$

### Performance evaluation

To measure the performance of applied link prediction approaches, we adopted the idea of precision@k^57,58^ or top $k$ predictive rate^53,59^. This metric is also known as $r$-precision^60–63^. precision@k is the recommended measure for link prediction algorithms^64^. It refers to the percentage of true positives among only the top $k$ ranked predicted links. Given the ranked output of the graph, we need to evaluate the ranking precision of the methods.

Following^26^, we chose the top 1%, 2%, and 5% as the value of $k$. In general, the area under the receiver operating characteristic curve (AUROC) or (AUC) is used to evaluate the performance of classification models. Nevertheless, recent studies have shown that AUROC is unsuitable for evaluating the performance of link prediction algorithms^55,65–67^. Another statistical measure is the area under the precision-recall curve (PRC), which potentially provides a more robust assessment, especially when dealing with imbalanced datasets^68^. In this study, we used precision@top, AUC, and PRC as performance metrics.

In order to compute some of the measures, we had to derive true positives (TP), false positives (FP), true negatives (TN), and false negatives (FN). To perform this, we ranked the predicted links in descending order based on the rank score given by the link prediction methods. Then, we considered several thresholds as cutoff values. The starting threshold is the minimum score given by the link prediction methods. Then we increase by a step size of 0.1, which was selected to ensure sufficient granularity in computing the area under the curve. We repeated this step until the threshold value was the same as the maximum score given by the link prediction algorithm. For each specific threshold score, if we found that the known link in the test dataset matched with the newly predicted link, and the score is greater than the threshold, we considered this matching as a true positive (TP) for evaluative purposes. Given an unknown link, which does not match the test dataset, but was predicted by the link prediction algorithm, and the score is greater than the threshold, we consider the case a false positive (FP). Similarly, when we found a known link (same as the test dataset and in the newly predicted links), but the score was below the threshold, we consider this a false negative (FN). Lastly, when we found any unknown link with a score below the threshold, we assigned the sample as a true negative (TN). Using the TP, FP, TN, and FN we calculated the “precision@top-1%”, “precision@top-2%”, “precision@top-5%”, AUC, and PRC.

### Data splitting for testing

To evaluating the performance of link prediction algorithms, the test data is generated by excluding a collection of links from the full homogenous network. Our homogenous network contains drug-drug, food-drug, and food-food similarities. We split 30% of links randomly to make the test data set, while the rest of the 70% of links are used for the training dataset. For stability, we repeat this evaluation ten times and report average performance.

### Ground-truth evaluation using DDS

Contrary to food-protein interactions^26^, there is no accessible gold standard for widely confirmed food-drug interactions. Therefore, we resorted to the extracted drug-drug interactions from DrugBank for ground truth evaluation. Since the graph representation in FDMine is homogenous (i.e., all nodes are chemicals), we can consider any part of the graph as a representative set of evaluation. Here, we remove 30% of the drug-drug links in the graph. Then, we execute the framework and report top-ranked cases for the precision evaluation. We split 30% DDS links (randomly) for making the test data set, while the rest of the 70% DDS, and all FDS, FFS links are used in the training dataset. Here, we measured the precision in terms of recovering the original links in the DDS subgraph. It should be noted that we also performed an evaluation using a random subset of any type of links (see Results).

We have performed three types of evaluations to benchmark the results. In the first evaluation, a drug can have a link with another drug based on similarity scores. In the second evaluation, a drug will have a correct link with another drug only if it is reported in the DrugBank database. The difference between the second and third evaluations is that the original links in the second evaluation are assumed based on the established similarity measures. Both evaluations will help us establish a comprehensive overview of link recovery in general and the validity of these recovered links using DrugBank. Although drug-drug interactions are examined in these two evaluations, they both provide estimates for the accuracy of food-drug predictions since the graph is homogenous in nature. The following Table 2 lists all the evaluative approaches we have performed in this study.

**Table 2:** List of evaluation approaches.

Title	Evaluation	Graph	Correct predictions	Methods
Evaluation 1	Remove random 30% of links from the DDIs (repeat 10 times)	Comprehensive evaluation for recovery of DDS similarity links	Match predicted links with the actual ones	All methods are applied
Evaluation 2	Remove random 30% of links (repeat 10 times)	Ground Truth using DrugBank	Match predicted links with DrugBank reported interactions	SP_2 (the best from evaluation 1 over disjoint graph) and RA (the best from evaluation 1 over joint graph)
Evaluation 3	Remove random 30% of links (repeat 10 times)	Whole graph including DDS, FDS, FFS	Match predicted links with the actual ones	SP_2 (the best from evaluation 1 over disjoint graph) and RA (the best from evaluation 1 over joint graph)
Evaluation 4	Prepare a list of gold standard food-drug interactions extracted from the literature. These interactions will be hidden from any training and will be used to measure and evaluate the validity of FDMine	Whole graph including DDS, FDS, FFS	Match predicted links with the actual food-drug interactions (gold standard dataset)	SP_2 (the best from evaluation 1 over disjoint graph) including SP_3 and RA (the best from evaluation 1 over joint graph) including AA, CN, and L3

### Implementation

We have deployed the code and run all experiments on a server with 64 GB of RAM, and Intel(R) Core(TM) i9-7980XE CPU @ 2.60 GHz (18 Cores, 36 Threads). For each run, the disjoint graph with shortest path and neighborhood algorithms took about 15 min and 1.7 h, respectively. The joint graph with the shortest path and neighborhood algorithms took 22 min and 2.3 h, respectively. For DrugBank data preprocessing, we used the Compute Canada cluster, and to calculate SSP, we used Google Colaboratory (a product from Google Research).

### Our proposed FDMine framework

**Figure 1:**
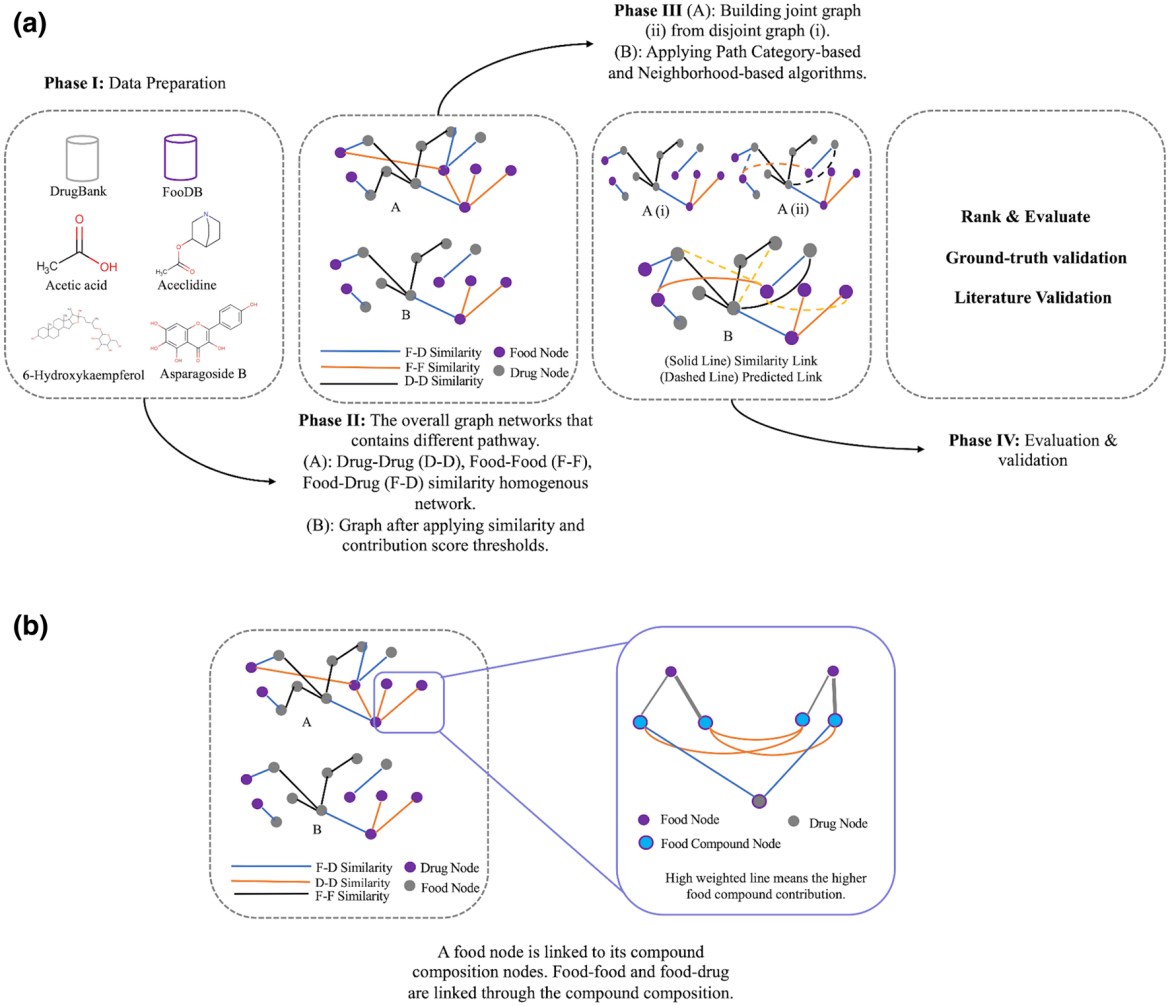
(**a**) The framework of FDMine. The main steps are I) preparing a comprehensive dataset describing FDIs by analyzing the whole DrugBank and FooDB databases with a unique representation of food composition II) defining a scoring function for computing chemical compound contribution in food items, III) implementing a set of path category-based (path length 2 and 3) and different neighborhood-based similarity-based algorithms to discover new FDIs from two different homogenous (disjoint and joint) graph networks, and IV) used the precision@k metric and calculated the precision@top (top 1%, 2%, and top 5%) for drug-drug links to verify the accuracy of the algorithms with the test dataset. (**b**) illustrates a zoom-in view of food-drug interactions such that food items are represented as nodes that are then linked to their composition nodes. The structural similarity is between the small molecule drugs and the food composition nodes. An aggregation step is applied to compute the similarity of food-drug based on the composition and contribution. This figure was generated using MS PowerPoint v16.54.

The FDMine framework (see Fig. 1) is composed of several phases. In Phase 1, raw data is parsed from DrugBank and FooDB databases. In Phase 2, we execute two steps including a) building a homogenous network based on the structure similarity profile, and b) updating the weights of the homogenous network using food compound contributions. Next, the graph is prepared with nodes representing drugs, food and food compounds’ composition. In the graph, links are weighted by similarity and contribution scores (see Phase 3 in Fig. 1). When applying the similarity thresholds, the homogenous network produces multiple subgraphs (disjoint graph). We build another version called the joint homogenous graph network and consider executing several link prediction algorithms, including applied path category-based and neighborhood-based similarity-based approaches. In the final Phase 4, we rank the newly predicted link (based on the score given by our methods), test the performance of the applied methods with the test dataset, and finally, consult the literature to validate the top FDIs found using the different methods. For testing, we perform a comparison using ground-truth and report literature validation for our leading findings (see “Results and Discussion” section).

## Results and discussion

The next subsections describe in detail the performance evaluation of FDMine, and the analysis of our novel FDI predictions.

### Prediction results of FDMine

#### Evaluation 1: comprehensive evaluation for the recovery of DDS similarity links

As explained earlier, DDS similarity links are a priority in our evaluation setup as it establishes a ground truth evaluation (see Evaluation 2 results). Here, drug-drug links are based on the similarity scorings we computed. We have applied two different link prediction approaches over two different types of homogenous graph networks. One is the disjoint graph network, and the other is the joint graph network. The applied methods are the path category-based and neighborhood-based similarity-based link prediction algorithms. We used path lengths 2 and 3 for the path category-based algorithm. SP_2 and SP_3 are used to describe (Path length 2), and (Path length 3), respectively. For neighborhood-based similarity-based link prediction, we applied Adamic and Adar Coefficient (AA), Common Neighbor (CN), Jaccard Index (JAC), Dice Coefficient (Dice), Resource Allocation (RA), and Multiple paths of length *l* = *3 (L3)*.

In our supplementary file, Table S4 provides a summary of different models over the disjoint graph network. For the disjoint graph, the SP_2 outperformed other methods. The precision rate for the top 1% (i.e., precision@top-1) is 84% for SP_2 while RA, the second-best has achieved 64%. For precision@top-2, SP_2 achieved the best results with 60% and *L3*, the second-best 42%. The highest value for the precision@top-5 was achieved by the SP_2 (40%). In the disjoint version of the graph, neighborhood-based similarity-based methods achieved, on average 17% with varying standard deviation each. However, SP_3 consistently demonstrated poor performance (5%, 3%, 2% for precision@top-1, precision@top-2, and precision@top-5, respectively) compared to all other methods. SP_2 achieved 52% and 26% AUC and PRC, respectively. All neighborhood-based similarity-based methods achieved more than 80% (AUC) except *L3* which had a reported precision of 60%. The PRC scores of the RA, AA, and CN were 70%, 65%, and 60% respectively.

**Figure 2:**
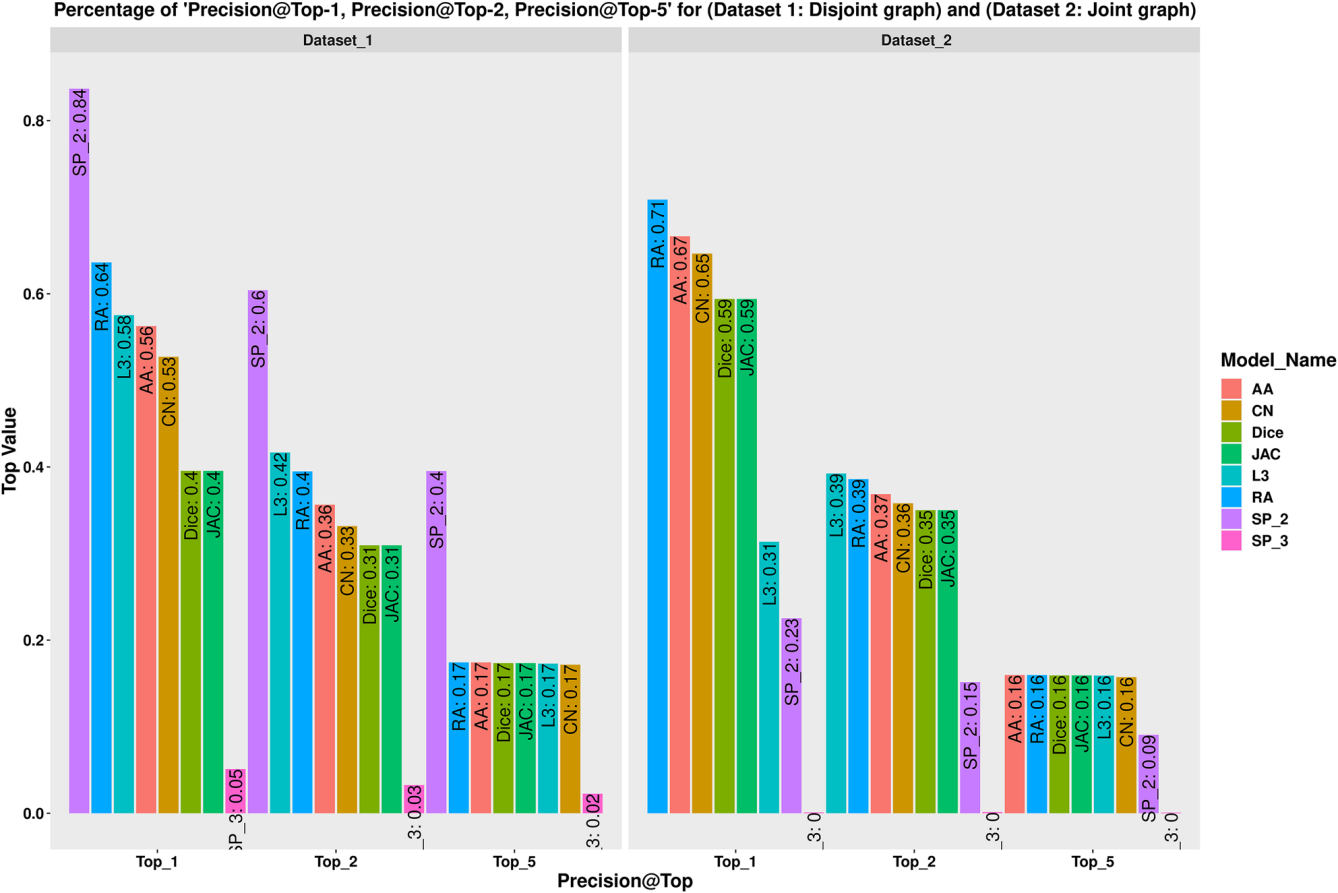
Comparison of the precision@top over eight methods and two different graph networks. This figure was generated using ggplot2 library from R v3.6.3.

When considering the joint version of the graph, different results were attained. The neighborhood-based similarity-based methods showed best results for the top precision@top-1, precision@top-2, and precision@top-5. For the precision@top-1, the RA achieved the best result (71%), followed by AA (67%). For the precision@top-2, *L3* and RA both yielded similar performance (39%). Additionally, all neighborhood-based similarity-based methods produced the same result (16%) for precision@top-5. Contrary to the case of the disjoint version of the graph, the performance of SP_2 was weak. The SP_2 achieved, 23%, 15%, and 9% for the precision@top-1, precision@top-2, and precision@top-5, respectively (see Tables S18–S20 for detailed results using 10 different random seeds). For the joint graph, the neighborhood-based similarity-based algorithms achieved AUC of more than 90% except L3 (65%). The value of the PRC is also high for the neighborhood-based similarity-based methods. The PRC scores for the RA, AA, CN were 87%, 86%, and 84% respectively. However, SP_3 always (disjoint and joint graphs) showed the weakest results in terms of all performance metrics (precision@top, AUC, and PRC). In Supplementary file 1: Table S5 summarizes the different models over the joint graph network. The comparison graph for the precision@top-1%, precision@top-2%, and precision@top-5% are provided in Fig. 2. For more details, see Supplementary file 1 Figures S7 and S8.

#### Evaluation 2: ground truth evaluation using DrugBank

The dataset we constructed using DrugBank and FooDB contains drug-drug links. The disjoint and joint dataset contains 2926, and 6581 drug-drug links, respectively. From evaluation 1, out of 2926, and 6581, our method managed to discover 1706, and 4178 of those links respectively, reported as DDIs in the DrugBank. We have considered these 1706 and 4178 as known DDIs and as ground truth. To cross-validate the performance of FDMine, we excluded a portion of known DDIs (or ground truth) as a test dataset from the main dataset and the rest of the dataset was used to train the models. Then, we calculate the precision@top-1%, precision@top-2%, and precision@top-5% and found approximately the same performance of FDMine with the disjoint dataset, and slightly better results for the joint dataset. Here, we have chosen only the best models, SP_2 for the disjoint dataset and RA for the joint dataset. Tables 3 and 4 provides the performance of FDMine with the ground truth test dataset.

**Table 3:** Performance evaluation of ground truth using disjoint dataset and path category-based (path length-2) method.

Method	Proportion	#Test DDI	#Matched DDI	Precision@ Top-1 (%)	Precision@ Top-2 (%)	Precision@ Top-5 (%)
SP_2	0.6	1023	864.8 (± 13.85)	84.49 (± 5.09)	72.29 (± 6.59)	47.11 (± 4.00)
	0.5	853	750.7 (± 9.91)	78.21 (± 7.50)	64.73 (± 4.86)	42.20 (± 2.79)
	0.4	682	613.5 (± 6.06)	76.31 (± 5.77)	57.51 (± 5.53)	36.81 (± 3.88)
	0.3	511	469.1 (± 4.93)	60.60 (± 9.06)	43.69 (± 5.44)	28.09 (± 2.57)

**Table 4:** Performance evaluation of ground truth using joint dataset and Neighborhood-based Similarity-based (RA) Method.

Method	Proportion	#Test DDI	#Matched DDI	Precision@ Top-1 (%)	Precision@ Top-2 (%)	Precision@ Top-5 (%)
RA	0.6	2506	2413.0 (± 9.12)	94.93 (± 0.30)	93.16 (± 0.71)	51.55 (± 0.71)
	0.5	2089	2027.4 (± 12.01)	95.99 (± 0.35)	86.64 (± 1.29)	40.63 (± 1.01)
	0.4	1671	1628.4 (± 6.97)	96.75 (± 0.49)	72.15 (± 1.07)	31.64 (± 0.54)
	0.3	1253	1223.3 (± 4.18)	90.96 (± 1.05)	54.59 (± 0.86)	22.97 (± 0.43)

Results are based on the Tanimoto coefficient $TC \ge 0.6$. We report performance over a different range of values in Supplementary File 1: Tables S6 and S7 for the disjoint and joint datasets, respectively.

#### Evaluation 3: prediction results for whole graph (DDS, FFS, FDS)

Here we randomly assigned 30% of all (DD, FF, FD) links from the whole dataset to make the test dataset, and the rest of the 70% was used to train the model. We applied ‘shortest path length 2’ over the disjoint and ‘RA’ over joint graph. The 30% test dataset from the disjoint and joint dataset contains 26,157 and 27,612 links respectively. FDMine was able to recover an average of 9612.6 (± 5723.06) and 27,448.4 (± 14.20) links from the disjoint and joint datasets using ‘shortest path length 2’ and ‘RA’ methods, respectively.

#### Evaluation 4: evaluation with gold standard dataset

Although Evaluations 1–3 reveal different aspects of the validity of FDMine, we considered reviewing the literature and built a list of well-reported food-drug interactions. We refer to this list of known food-drug interactions as the Gold Standard dataset. These interactions are fully hidden from the inference steps in FDMine and will only be used to evaluate the accuracy of the model. Details on the steps used and cases prepared for the dataset can be found in Supplementary File 1 (see 15. Details of the Gold Standard Dataset). For example, several studies referred to the interaction of Warfarin and Vitamin K^69,70^. In this evaluation, we will test if FDMine can reveal these known interactions. All steps for data preparation and thresholds are the same as the previous evaluations. This gold-standard dataset contain 53 FDIs.

FDMine recovered 46.15% and 44.23% from the joint datasets using ‘L3’ and ‘SP_L3’ respectively. The recovery rate for the ‘SP_L2’, ‘RA’, ‘AA’, and ‘CN’ was 5.77%. These results illustrate the advantage of introducing joint links and using longer path length (i.e., path length of 3) in matching ground-truth FDIs. The detailed findings are given in the Supplementary file 1: Tables S8 and S9 for the disjoint and joint datasets respectively.

### New food drug interaction prediction

After comparing the different approaches for link prediction, we executed the FDMine framework to find top candidates for FDIs. In the framework, we consider taking the top results from the joint and disjoint versions. At the final stage of FDMine, we surveyed the literature to find supporting evidence to the generated predictions. We have performed two batches using different contribution scores (i.e., 0.5 and 0.3, respectively). The default value in the FDMine framework is a 0.5 contribution score. As listed in Supplementary file 1, the results have demonstrated some repeated drugs among the top findings associated with a higher threshold value. A high threshold value will lead to removing more connections in the graph. This will lead to more disjoint subgraphs, and nodes with higher connections within the subgraphs exhibiting higher rank scores. Therefore, we consider a more relaxed threshold and generate Batch-2 results (i.e., contribution score of 0.3). In this batch, we see more diversity in our results. Supplementary file 1 (see 16. Batch-1 Description and Result) lists all Batch-1 results, and Supplementary file 1 (see 17. Batch-2 Description and Result) lists all Batch-2 results with a description of the experiments used in each. We analyzed all results of both batches and discussed the insights driven from two types of evidence: (1) linking food to anti-inflammatory effects based on known biological pathways and (2) linking food to pharmacological effects based on matching functions of a drug and a chemical substructure found in food.

### Food compound compositions with anti-inflammatory effects (biological pathway driven evidence)

**Figure 3:**
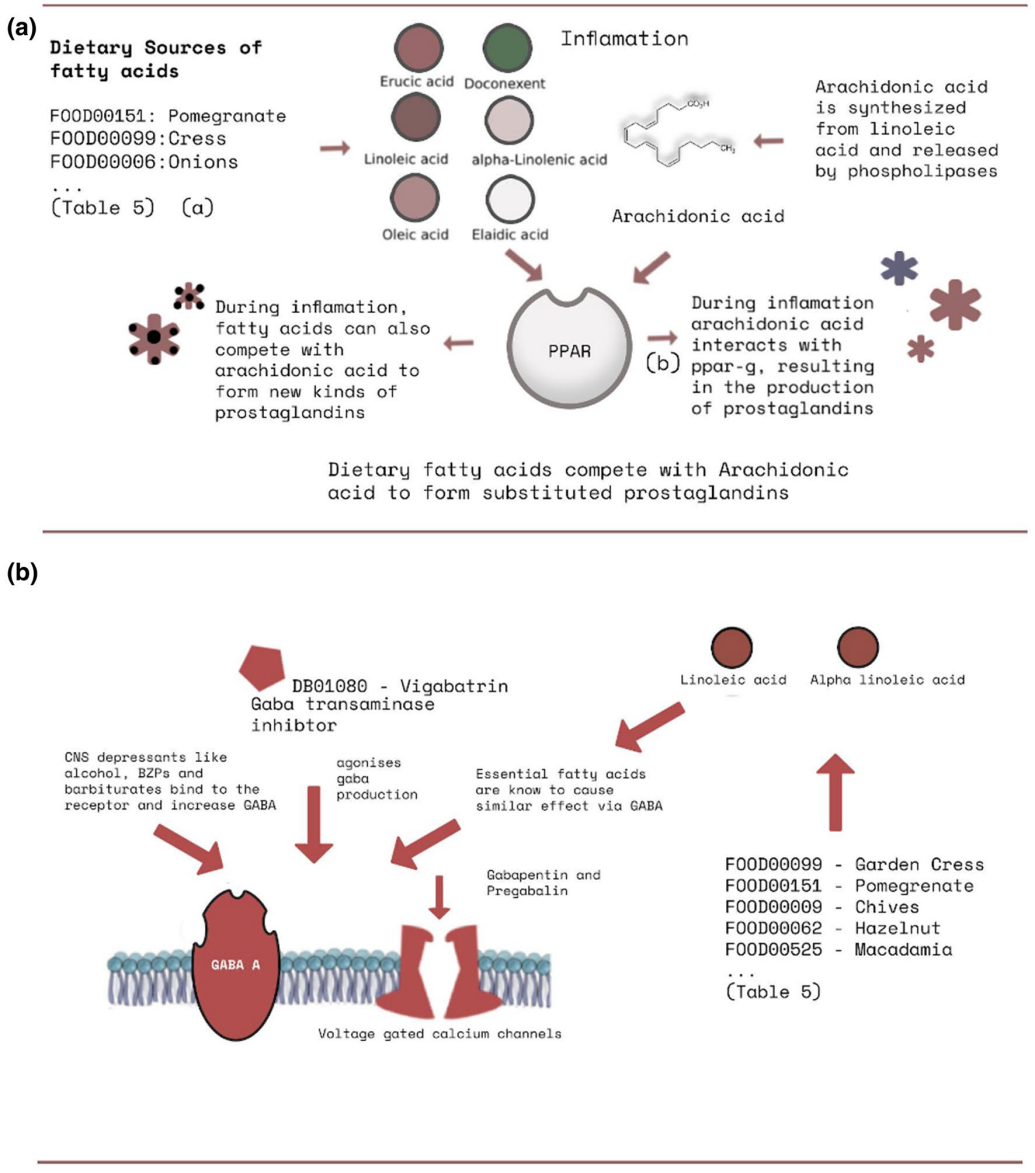
(**a**) An illustration depicting the effect of dietary fatty acids on COX pathway a) Various foods are rich sources of dietary fatty acids (**b**) During inflammation, Arachidonic acid interacts with PPAR to produce prostaglandins (**c**) Dietary Fatty acids can compete with Arachidonic acid during inflammation at PPAR to form substituted prostaglandin variants. (**b**) An illustration depicting Gabaergic drug mechanisms. Dietary sources containing fatty acids increase the production of GABA. Taking drugs like Vigabatrin, pregabalin & Gabapentin with such a diet can increase Gabaergic effects. This figure was generated using Photopea.com online graphics editor.

The results in this section are part of Batch-1 results (see Supplementary file1: 16. Batch-1 Description and Result). Our findings using a literature review indicate possible pairings of drug and nutraceutical food components. As per the summary in Table 5, the interactions we obtained appear to affect key biological pathways including—prostaglandin biosynthesis for inflammatory response^71^, beta-adrenergic signaling for cardiac output modulation^72^ and GABA pathway^73^—a GABA based inhibitory neurotransmitter that down-regulates central nervous system stimulation^74^. After examining the results in Table 5, we have found that dietary fatty acids like Oleic acid (FDB012858), Erucic acid (FDB004287), (Z,Z)-9,12-Octadecadienoic (FDB012760) and Elaidic acid (FDB002951), available in foods like Onions—FOOD00006, Garden Cress—FOOD00099, Pomegranate- FOOD00151, etc., can affect prostaglandin biosynthesis via peroxisome proliferator-activated receptor (PPAR) mediated mechanism and Gabaergic pathway. Figure 3a,b highlight the list of these compounds and their interaction with PPAR and GABA-mediated effects, respectively. Similarly, we found evidence of food components like Eugenol (FDB012171), Carvacrol (FDB014512), which can potentially substantiate hypotensive effects when taken with beta adrenergic drugs. For example, Eugenol has been known to cause vasodilation via vanilloid TRPV4 receptors found on endothelial muscles in arteries. Beta-adrenergic drugs are prescribed to patients suffering from hypertension to decrease blood pressure (BP). So, when combined, this can cause an increased reduction in BP.

Prostaglandins are compounds that play a role in the anti-inflammatory pathway during injury^75^. An essential molecular building block in humans is arachidonic acid. It interacts with the peroxisome proliferator-activated receptor (PPAR) to form various prostaglandins^75^ or anti-inflammatory compounds. Various dietary fatty acids (see Table 5; Oleic acid, Linoleic acid, Erucic Acid, Eldaic acid) are also absorbed via the exogenous chylomicron pathway and hydrolysed for various tissues to absorb them for further processing^76^. Some of our predicted compound items include Oleic acid—FDB012858, and Erucic acid—FDB004287, that are similar to arachidonic acid and are analogous^77^ structures, belonging to the fatty acid group and are found in many dietary sources including Celery—FOOD00015, Peanuts (FOOD00016) and Burdock—FOOD00017 (see Table 5). Our literature review has highlighted reported evidence on the influence of these dietary fatty acids on the arachidonic acid cycle. Arachidonic acid is a precursor for the synthesis of various other biomolecules, associated with anti-inflammatory pathways^78^. During injury, inflammation occurs and causes arachidonic acid to bind with PPAR-gamma receptors as shown in Fig. 3a to form prostaglandins or protective anti-inflammatory agents to curb injury^79^. Fatty acids (see Table 5) also compete with arachidonic acid during injury or inflammation to produce various substituted prostaglandins belonging to a family of derivative compounds known as eicosanoids^80^, via PPAR^81^. Since the substituted prostaglandins are not exactly derived from arachidonic acid, they show slightly fewer anti-inflammatory profiles than other eicosanoids produced directly from arachidonic acid^82^. It is worth noting that arachidonic acid belongs to the list of essential fatty acids, including alpha-linoleic acid and docosahexaenoic acid^82^. There has been evidence to show that dietary sources such linoleic acid, erucic acid and elaidic acid (see Table 5) did increase PPAR gene expression in healthy subjects^83^. In 2012 Hung-Tsung Wu et al. also showed the interaction of oleic acid with PPAR-g receptors^84^. These results may suggest that taking drugs like Doconexent—DB03756 with foods such as FOOD00099—Garden Cress, FOOD00151—Pomegranate, FOOD00009—Chives, FOOD00062—Hazelnut, FOOD00525—Macadamia, can alter the normal dynamics of anti-inflammatory responses. Arachidonic acid is biosynthesized from dietary linoleic acid and released by phospholipases during inflammation. This pathway is also known as the COX or Cyclooxygenase pathway^85^.

**Table 5:** Depicts some of our top correlations of food substances that can potentially be involved in food drug interactions when combined with a drug with similar activity.

Food component	Food source ID	Food name	Pharmacological actions	Drug	References	Batch
Oleic acid	FOOD00006	Garden Onion	Dietary fatty acids like Oleic acid can compete with arachidonic acid by interacting with PPAR-g receptor to form prostaglandins They can also cross the blood brain barrier and interact with GABA receptors to induce anxiolytic & possible anti-epileptic effects	Vigabatrin, Pregabalin, Gabapentin Doconexent	^81,84,86–89^	Top 10 in joint and disjoint—batch 1 (See Supplementary file 1: 16. Batch-1 Description and Result )
	FOOD00009	Chives				
	FOOD00011	Cashew Nus				
	FOOD00012	Pineapple				
	FOOD00015	Wild celery				
	FOOD00016	Peanuts				
	FOOD00017	Burdock				
	FOOD00021	Asparagus				
	FOOD00024	Brazil Nut				
	FOOD00026	Borage				
Erucic acid	FOOD00099	Garden Cress				
Elaidic acid	FOOD00151	Pomegranate				
(Z,Z)-9,12-Octadecadienoic acid	FOOD00009					
Eugenol	FOOD00179	Cloves	Eugenol causes vasodilation via vanilloid TRPV4 receptors found on endothelial muscles found on arteries. Eugenol & Capsaicin have a vanilloid ring. TRPV4 is involved in BP regulation via various mechanisms	Betaxolol, Atenelol, Esmolol, Bisprolol, Metoprolol	^85,90^	Top 20 in joint and disjoint—batch 2 (See Supplementary file 1: 17. Batch-2 Description and Result)
Isopropyl-2-methylphenol	FOOD00089	Hyssop	p-Cymene has been reported to cause smooth muscle vasodilation and has antihypertensive effects			
1-Isopropyl-4-methylbenzene	FOOD00013	Dill	Also known as p-cymene. It has been shown to cause sedative effects via GABA adrenergic receptors and also causes vasodilation of smooth arterial muscles			
1-Methoxy-4-(2-propenyl)benzene	FOOD00137	Anise	Methyl Chavicol has been reported as an adjunct therapy for treatment of hypertension, found in anise			
	FOOD00019	Tarragon				

Each food component can link to any drugs as long as they are in the same batch.

### Food compound composition with pharmacological effects (similar function-driven evidence)

Here, we relaxed the contribution score to 0.3 (i.e., Batch-2) to obtain a diverse set of results (Supplementary file 1: 17. Batch-2 Description and Result). In this part of our literature validation, we analyze the potential of similar functions of drugs and food compounds on specific diseases. The results in Table 5 highlight some correlations with a group of drugs called beta-adrenergic drugs and essential oils. Our top correlated pairs of food and drug observed that both of them caused reduced blood pressure. Beta-blockers are used to treat hypertension in patients. Beta-blockers consist of b1, b2, and b3 subtype receptors. Beta-blockers can fall into various categories based on the extent of selectivity of binding across these subtypes. For example, Atenolol (DB00335), Bisoprolol (DB00612), Metoprolol (DB00264) and Esmolol (DB00187) are b1 selective blockers^91^. The effects of b1 blockade include a decrease in cardiac output by inhibiting the SA and AV nodes, thereby decreasing stroke volume^86^. Propranolol (DB00571) and Penbutolol (DB01359), on the other hand, are non-selective beta-adrenergic blockers. Studies have also observed that beta-blockers may also contribute to GABA turnover in the cerebrum^87^.

The results suggest that beta-blocker drugs like Atenolol, Betaxolol, Esmolol, Oxprenolol, Penbutalol, and Propranolol can synergistically interact when combined with a specific compound composition including p-Cymene—FDB014512, Eugenol—FDB012171, and Carvacrol (terpenoid substances). For example, Marcio et al. 2011 reported that monoterpenoids like p-Cymene—FDB014512 and Carvacrol have vasorelaxant effects^85^.

We were able to confirm that fatty acids (Oleic acid (FDB012858), Erucic acid (FDB004287), (Z,Z)-9,12-Octadecadienoic (FDB012760) and Elaidic acid (FDB002951) ) can cross the blood–brain barrier and be beneficial to relieve anxiety^88^. They are also believed to act via stimulation of GABA-A based receptors. Benzodiazepines, barbiturates^89^ and some anticonvulsants act by modulating the GABA receptors^92^. The inhibitory effects of GABA help relieve seizures. However, drugs like Pregabalin and Gabapentin instead act by blocking calcium or sodium channels to help stabilize seizures. Although this is not directly interacting with GABA receptors, it helps reduce excitatory neurotransmitters. Thus, they may help substantiate antiepileptic activity by increasing amounts of GABA.

In summary, the discussed pairs of food ingredients and drugs can influence their own pharmacokinetics. For example, taking beta-adrenergic drugs with food containing terpenes like Eugenol and Methyl chavicol can potentially cause more pronounced antihypertensive effects. Taking antiepileptic medications and foods containing fatty acids can potentially elevate overall GABA levels beyond the levels achieved when taken individually. Moreover, dietary fatty acids can also interact with the PPAR receptor during inflammation to produce variations of prostaglandins. This demonstrates the feasibility of using our FDMine framework to identify potential food and drug interactions.

### Limitations and future work

There are some limitations associated with our current framework. For instance, FDMine did not consider the weight of the nodes (e.g., the degree of a node) to generate the joint version of the graph from the disjoint representation. Instead, FDMine used randomly chosen nodes to establish connections across disjoint groups. Additionally, the reported results illustrate that the precision@top has a significant drop for the SP_3 compared to the SP_2. In FDMine, an accumulated score that combines SP_2 and SP_3 was not introduced. This could have alleviated the drop in performance evaluation while taking advantage of the extra information gained from a longer path length when possible. Given the nature of a data-driven approach, an experimental validation would have helped further validate the findings of FDMine.

Future work will address various aspects of how FDMine can be improved upon. Currently, FDMine is evaluating the shortest path using the Dijkstra algorithm. One possible future research avenue would be to modify Dijkstra's algorithm to find the longest path by inverting the similarity scores. Another research option would be to assign different edge weights to generate the joint version of the disjoint graph. Similarly, the connection between two disjoint graphs can be made based on the degree of the node, e.g., maximum or minimum degree nodes can be chosen instead of randomly chosen nodes.

Moreover, the neighbourhood algorithms do not consider path weight. These algorithms function based on their mathematical formulation where the edge weight is missing. Here, we can modify this formulation so that these algorithms can incorporate the edge weight to produce FDMine’s results.

## Conclusion

In this study, we introduced FDMine as a framework to infer the interaction between food compounds and drugs using a homogenous graph representation. This homogenous representation enables us to take advantage of reported drug-drug interactions for accuracy evaluation, especially when accessible ground truth for FDIs is lacking. We considered several resources to construct food-drug, drug-drug, and food-food similarity profiles. FDMine uses established path category-based and neighborhood-based similarity methods to predict FDIs efficiently. A subset of Drug-drug interactions was used as ground-truth evaluations. This proposed methodology is based on encoding all entities including drug and food into a homogenous graph of chemical nodes. Therefore, any part of this graph can then be used as a representative evaluation, potentially informative to clinicians and researchers. We prepared a gold standard dataset from the well-referenced FDIs reported in the literature to perform external validation. Additionally, we have performed two types of evaluations to benchmark results using different parts of the graph. The shortest path-based method has achieved a precision 84%, 60% and 40% for the top 1%, 2% and 5%, respectively. FDMine was able to achieve an average 99.4% recovery rate from 27,612 available links in the joint version of the graph. For the gold standard evaluation, FDMine recovered 46.15% of the ground-truth cases from the joint datasets using a path length of size 3 and the neighborhood-based algorithm. We validated the top FDIs predicted using FDMine to demonstrate the applicability of the model. In the literature validation, we discussed the therapeutic effects of a group of food items. We observed that a set of FDIs may reduce blood pressure, have anti-inflammatory effects or reduce seizure. The benchmark results and literature review suggest that FDMine can help identify FDIs precisely and may represent an advanced strategy in drug discovery.

## Supplementary Information

Supplementary Information.

## Data Availability

The code and datasets supporting the conclusions of this article are included within the article (and its supplementary files) or is made available at https://github.com/mostafiz67/FDMine_Framework.
