# Supplementary material for: A novel graph mining approach to predict and evaluate food-drug interactions
Source: Sci Rep. 2022 Jan 20;12:1061. doi: 10.1038/s41598-022-05132-y (PMC8776972; doi:10.1038/s41598-022-05132-y)
Supplement: Supplementary file 1 — Supplementary Information. [file 41598_2022_5132_MOESM1_ESM.pdf]

A novel graph mining approach to predict and evaluate food-drug interactions

Md. Mostafizur Rahman[1], Srinivas Mukund Vadrev[1], Arturo Magana-Mora[2], Jacob Levman[1,*] and Othman Soufan[1,*]

[1]*Department of Computer Science, St. Francis Xavier University, Antigonish, Nova Scotia, Canada*

[2]*Saudi Aramco, EXPEC Advanced Research Center, Drilling Technology Team, Dhahran, 31311, Saudi Arabia.*

[*]*jlevman@stfx.ca*, [*]*osoufan@stfx.ca*

Supplementary File 1

1. DrugBank Dataset Extraction

The extraction technique for the DrugBank dataset is depicted in the data flow diagram (Figure S1). The primary DrugBank database is extensive and contains a wealth of data. We parsed the entire dataset with 'dbparser v1.2.0,'[*] a package of the 'R' programming language. Only the 'approved' drug group and 'small molecule' type drugs were explored in this project. We utilized the 'R' programming language package 'dplyr' to perform this task. Finally, we had 1,683 distinct approved small molecule drug information and 788 distinct approved molecule drug with metabolic related interactions after compressing the primary DrugBank dataset.

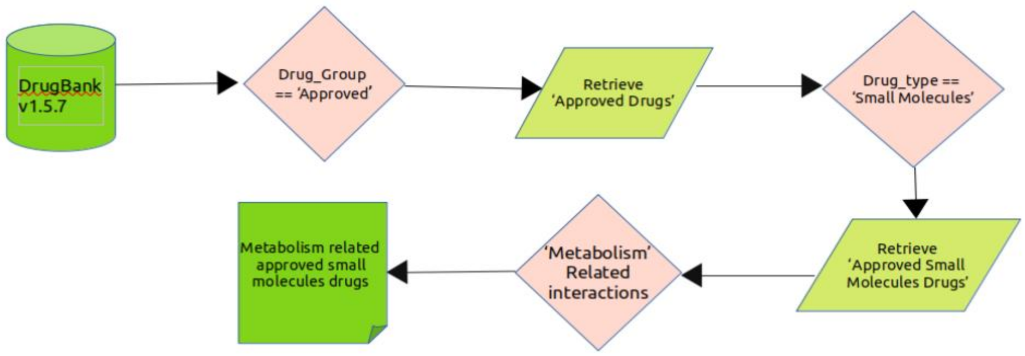

Figure S1: DrugBank Dataset Extraction Procedure. This figure was generated using MS PowerPoint v16

(*) Ali, M. & Ezzat, A.dbparser: 'DrugBank' Database XML Parser version 1.2.0 from CRAND ainanahan. https://rdrr.io/cran/dbparser/(2020).17

2. FooDB Dataset Extraction

We used the FooDB Version 1.0 dataset in JSON format to generate the food dataset. To parse the database for data pre-processing, we employed the 'R' programming language. For this project, we needed versions 1.3.0 and 1.0.5 of the "tidyverse" and "dplyr" packages, respectively. Figure S2 is a block diagram of the exclusion criteria we used to analyze food data.

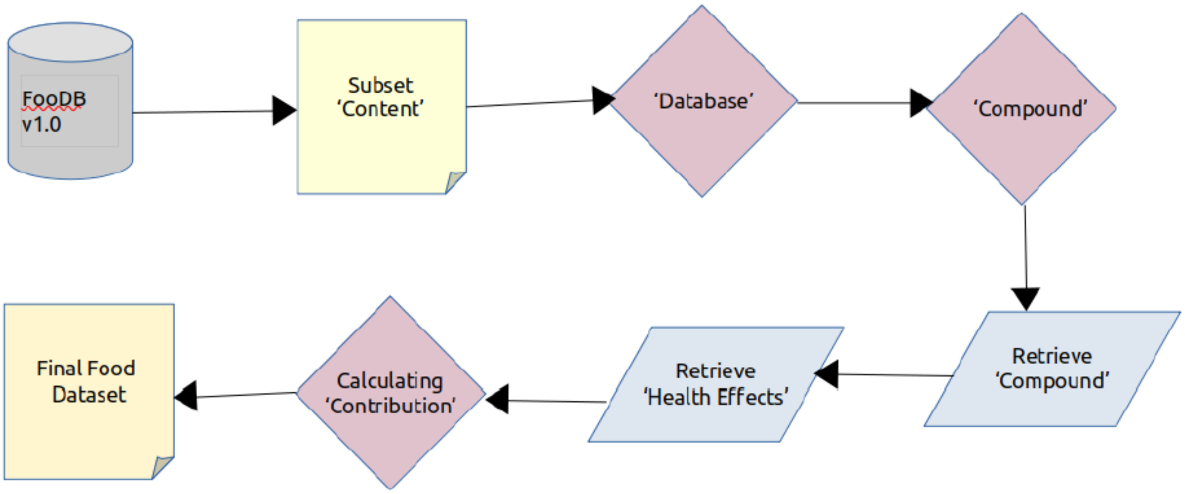

Figure S2: FooDB Dataset Extraction Procedure. This figure was generated using MS PowerPoint v16.

3. Structure Similarity Profile

A structural similarity profile (SSP) is a feature vector containing a distinct numerical representation, after accumulating structural features of individual food components and drugs. The SSP is a collection of paired structural similarity scores derived from a comparison of all 788 DrugBank approved small molecule drugs and 8,846 distinct food components. The Tanimoto coefficient was used to determine the structural similarity between two nodes (e.g.: food-drug, drug-drug, and food-food). Tanimoto coefficient is an efficient approach to figure out how similar two structures are based on their chemical fingerprints. The Tanimoto coefficient is calculated by comparing the number of common chemical fingerprints between the two drugs to the total number of chemical fingerprints. Morgan/Circular fingerprints were used to calculate the chemical fingerprints of each drug.

To compute the SSP of individual pairs, we utilized the Python package 'RDKit' (version 2020.09.2). To compute the SSP, we performed four steps:

Step-1: Examine the SMILES to see if they are correct. Because the core SMILES structure may have been destroyed or contains noise at the time of parsing.

Step-2: Prepare a set of mols using the SMILES structures.

Step-3: Prepare a set of fingerprints (fp) from the mols.

Step-4: Evaluate all fp pairwise without creating any duplicates. In this case duplicate means, we did not check the same drug-drug and food-food fingerprints. This avoids creating drug-drug and food-food pairings with the highest possible score (e.g.: 1).

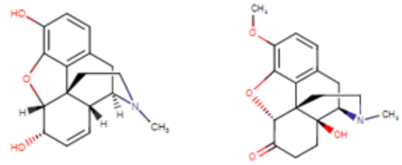

Morphine Oxycodone

Morphine's fingerprint	1	0	1	0	…	0	1	0
Oxycodone's fingerprint	0	0	1	0	…	0	1	1

Tanimoto Coefficient (Morphine, Oxycodone)

$$= \frac{|\,\text{Morphine} \cap \text{Oxycodone}\,|}{|\,\text{Morphine} \cup \text{Oxycodone}\,|}$$

Figure S3: Calculating Structure Similarity Profile Using Tanimoto Coefficient. This figure was generated using MS Word v16.

4. Food Compound's Contribution

We calculated the food compound contribution using the given formula. A food compound's contribution has a huge impact in predicting food-drug interactions. In the Table S1, this food contains 4 different compounds. All of these compounds do not have the same contribution. So, it would not be appropriate to use the score from SSP directly in the models. So, we use the contribution of the food compound to predict FDIs efficiently.

$$Contribution\ score(normalized) = \frac{Compound\ original\ content \in a\ food\ item}{Total\ original\ content\ of\ all\ compounds \in a\ food}$$

Table S1: Calculating the contribution of a food compound in a food

Food_ID_Compound_ID_Compound_Name	Original Content of a Compound in the food	The total original content in the food	Contribution of the compound in the food (0 ~ 1)	Contribution of the compound in the food (%)
FOOD00005 _ FDB000633 _ Kaempferol	4.10000	6.500	0.630769230769231	63.0769230769231
FOOD00005 _ FDB002602 _ Cyanidin	0.00000	6.500	0.000	0.00
FOOD00005 _ FDB002798 _ Apigenin	0.00000	6.500	0.000	0.00
FOOD00005 _ FDB011904 _ Quercetin	2.40000	6.500	0.369230769230769	36.9230769230769

5. Disjoint and Joint Graph

When a graph dataset contains multiple subgraphs, this is known as disjoint graph network. Our main dataset contains disjoint graph networks. Over this disjoint graph network, we applied eight different link prediction algorithms. After that, we created a joint graph network from the same disjoint graph network. To make the joint graph network, we chose any node (randomly) from the subgraphs of the disjoint graph network and add an edge among all subgraphs. Also, we gave a very small edge weight 0.00001 to the new connected edge.

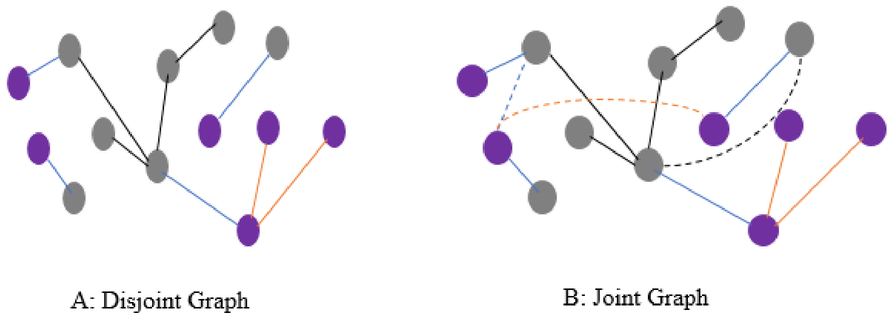

Figure S4: Disjoint and Joint Graph. This figure was generated using MS PowerPoint v16

In the Figure S4, the left network (A: Disjoint graph) contains 3 subgraphs. To make the right network (B: Joint graph), we chose any node from the available subgraphs and connect them with 0.00001 edge weight. The dashed line indicates the new edge created to convert the disjoint graph into a joint graph.

6. Path Category-based Algorithm

A 2-length path, such as "Drug1-Food1-Food2" (see Figure-1) connects the Drug1 node with the Food2 node through the similarity between "Drug1 and Food1" and "Food1 and Food2". This is defined as a D-F-F path. As illustrated in Supplementary file 1: Fig-1, the gold color circle denotes the food node and silver color circle denotes the drug node. There are 8 possible combinations of paths (i.e., Drug-Drug-Drug, Drug-Food-Drug, Food-Food-Food, Food-Drug-Food, Drug-Drug-Food, Drug-Food-Food, Food-Drug-Food, and Food-Food-Food).

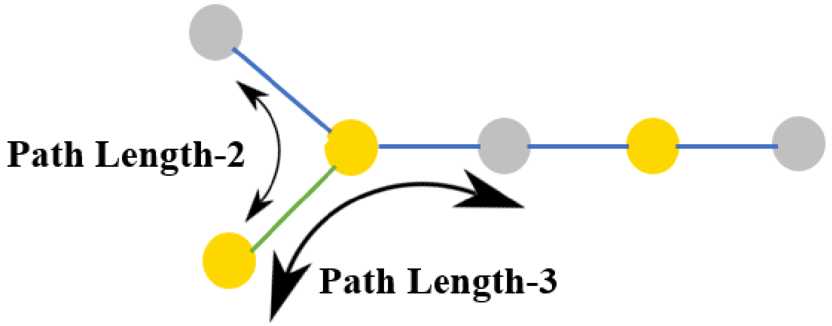

Figure S5: Example working procedure of the Path Category-based method. This figure was generated using MS PowerPoint v16

7. Precision@Top

We have evaluated FDMine with precision@k. We calculated the top 1%, top 2%, and top 5% for our values of *k*, based on the score given by link prediction algorithm. For example, for the following Figure S6, we have 10 new links. So, top 1% is the first entry (Link-1). We are able to match the first link. So, the precision@top-1 is 1. Again, for top-2, we have 2 links (Link-1 and Link-2). Between these 2 links we are able to match 1 and unable to match 1. So, precision@top-2 is 0.5. Same for top-5.

Newly Predicted Link	Score given by model
Link-1	1
Link-2	1
Link-3	0.9
Link-4	0.9
Link-5	0.7
Link-6	0.6
Link-7	0.5
Link-8	0.5
Link-9	0.4
Link-10	0.2

Figure S6: Precision@Top

8. Comparison of the Precision@top rate over eight link prediction methods and two different graph networks

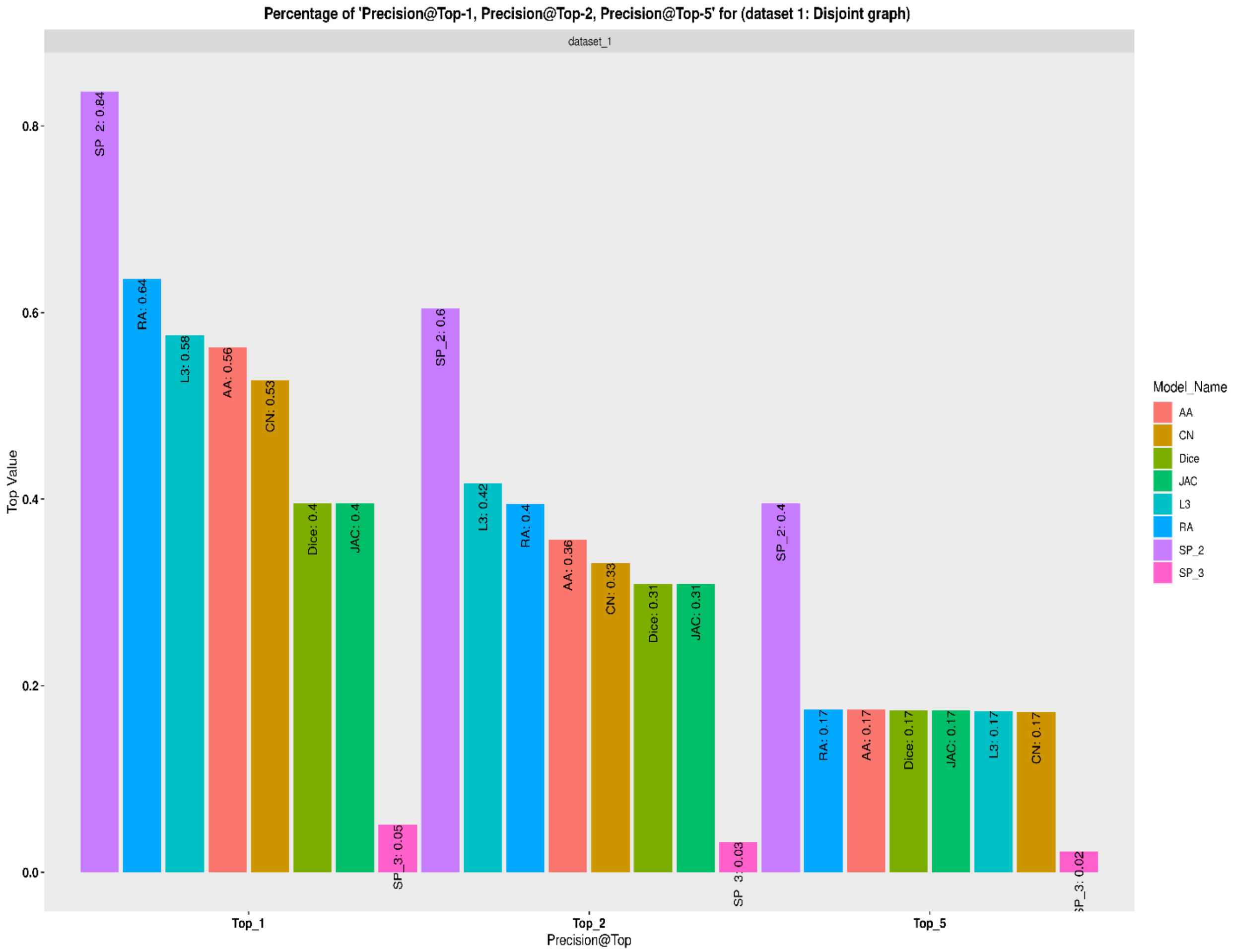

Figure S7: Precision@top comparison of eight different methods over the disjoint graph network. This figure was generated using ggplot2 library from R v3.6.3.

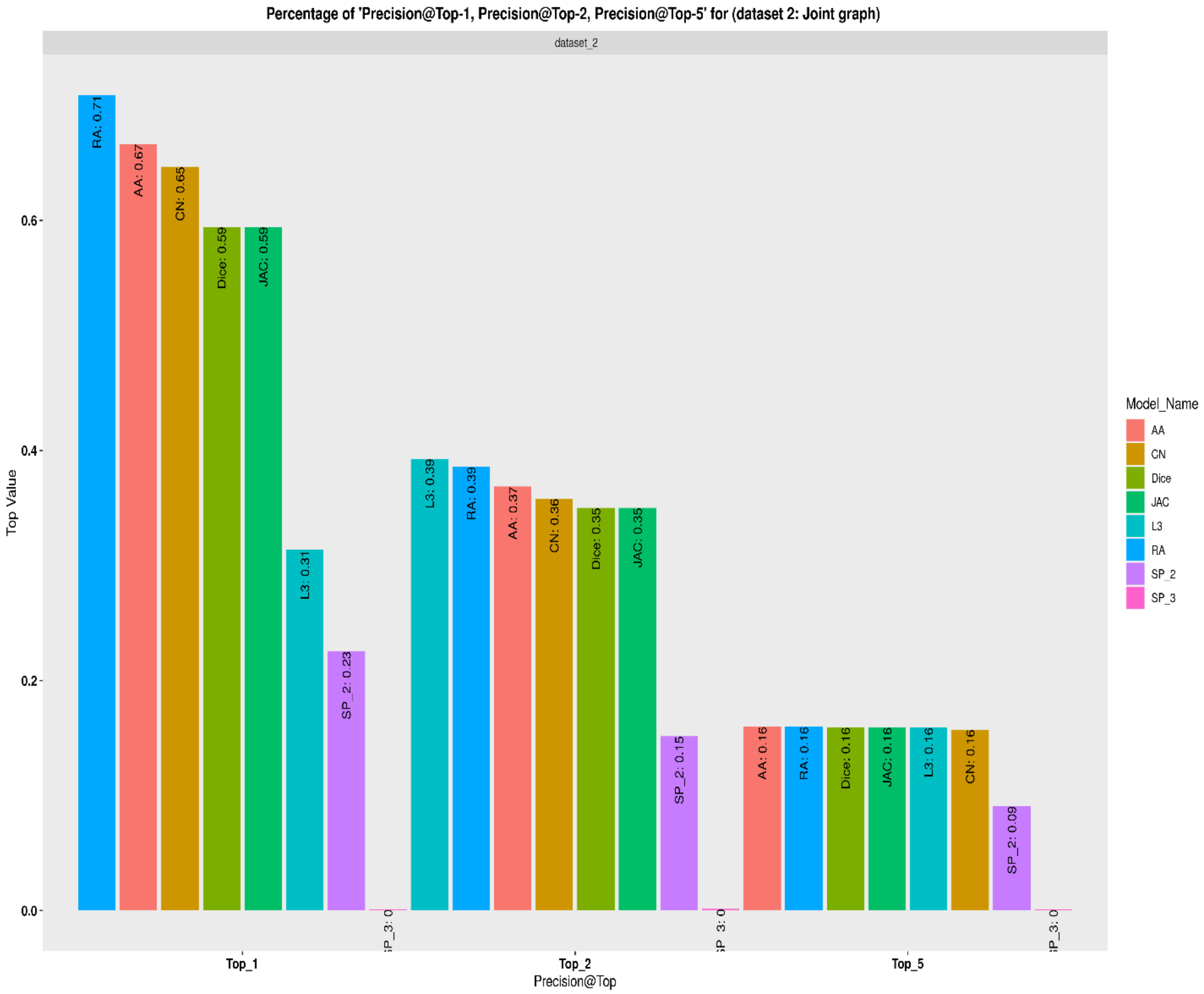

Figure S8: Precision@top comparison of eight different methods over the joint graph network. This figure was generated using ggplot2 library from R v3.6.3.

9. Area Under the Curve (AUC)

To evaluate the performance of the link prediction models we used AUROC (Area Under the Receiver Operating Characteristic Curve). This is one of the most important evaluation metrics. To calculate AUROC, we need the true positive rate (TRP) and false positive rate (FPR). TRP and FRP calculated based on the formula given below:

$$True\ Positive\ Rate\ (TRP) = \frac{True\ Positive}{True\ Positive + False\ Negative}$$

$$False\ Positive\ Rate\ (FPR) = \frac{False\ Positive}{True\ Negative + False\ Positive}$$

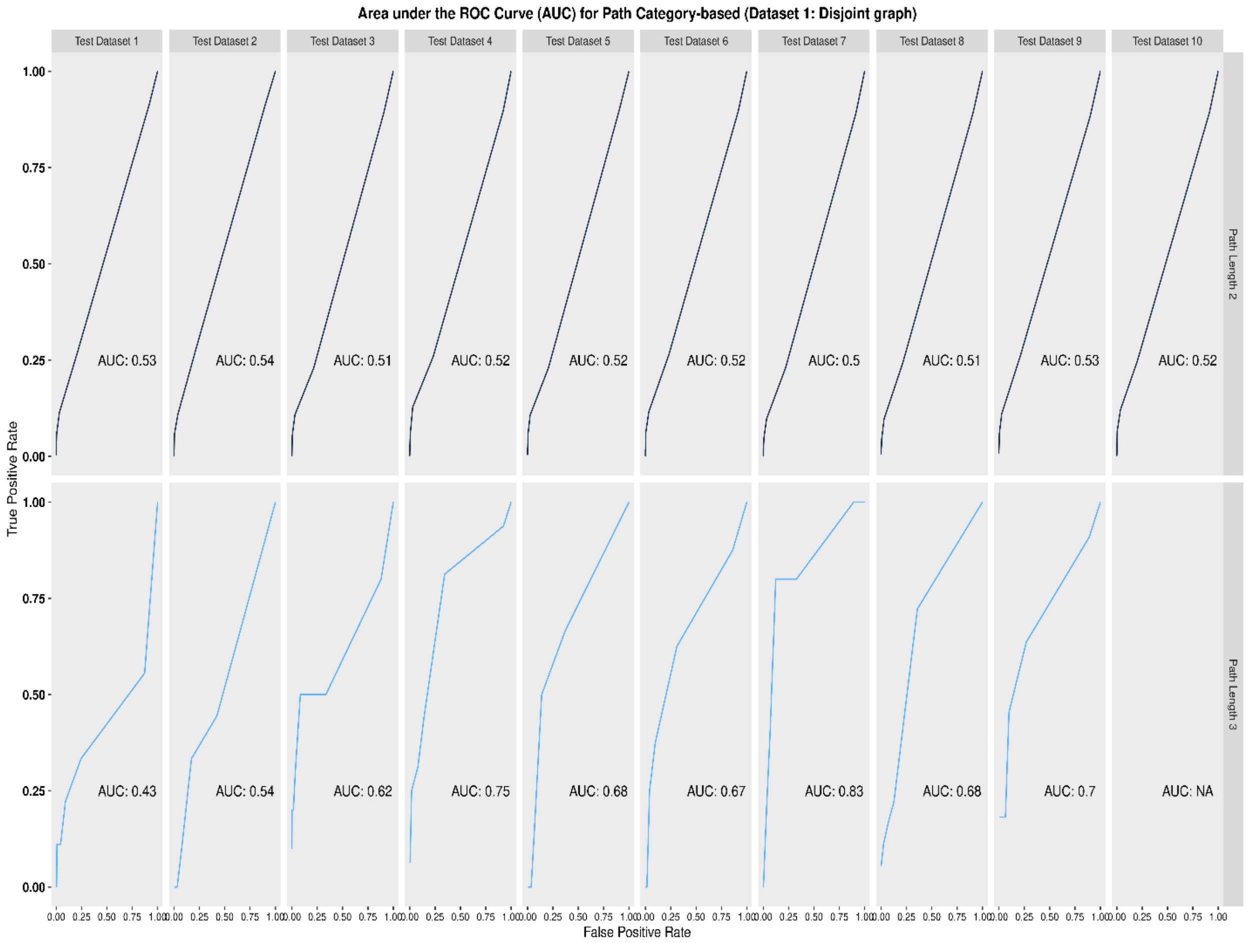

Figure S9: Area Under the Curve (AUC) for path category-based (dataset 1: disjoint graph). This figure was generated using ggplot2 library from R v3.6.3.

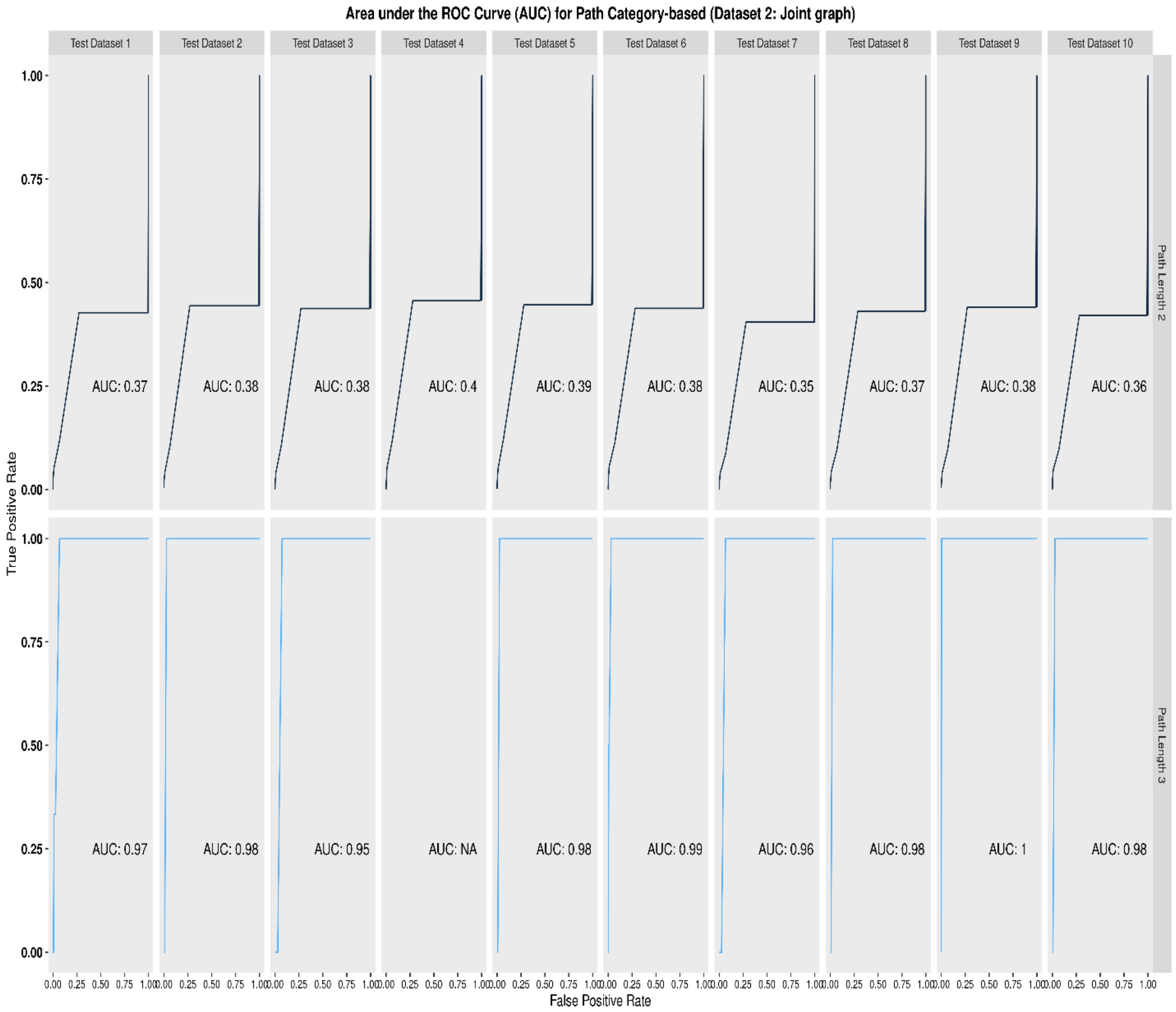

Figure S10: Area Under the Curve (AUC) for path category-based (dataset 2: joint graph). This figure was generated using ggplot2 library from R v3.6.3.

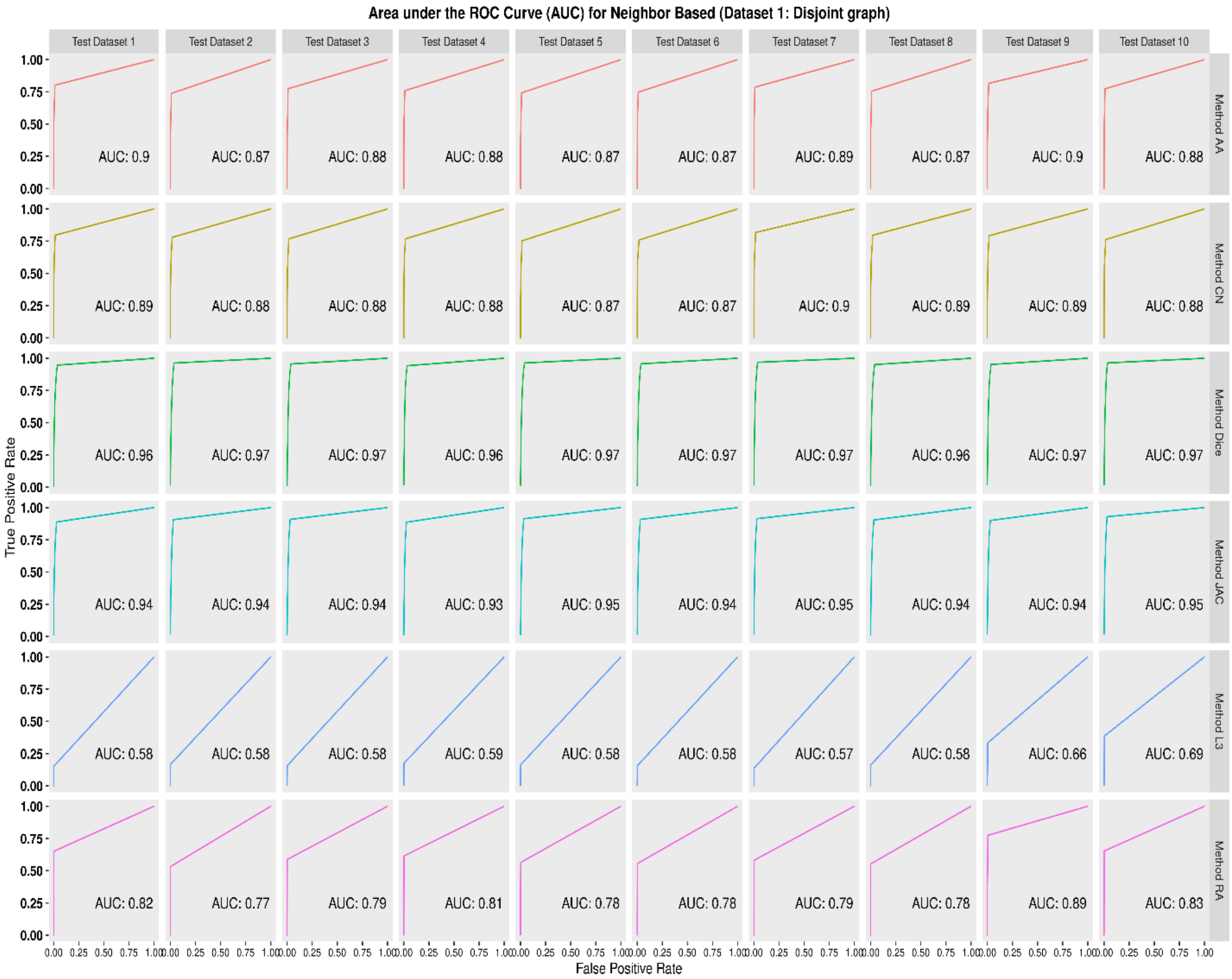

Figure S11: Area Under the Curve (AUC) for neighborhood-based similarity-based (dataset 1: disjoint graph). This figure was generated using ggplot2 library from R v3.6.3.

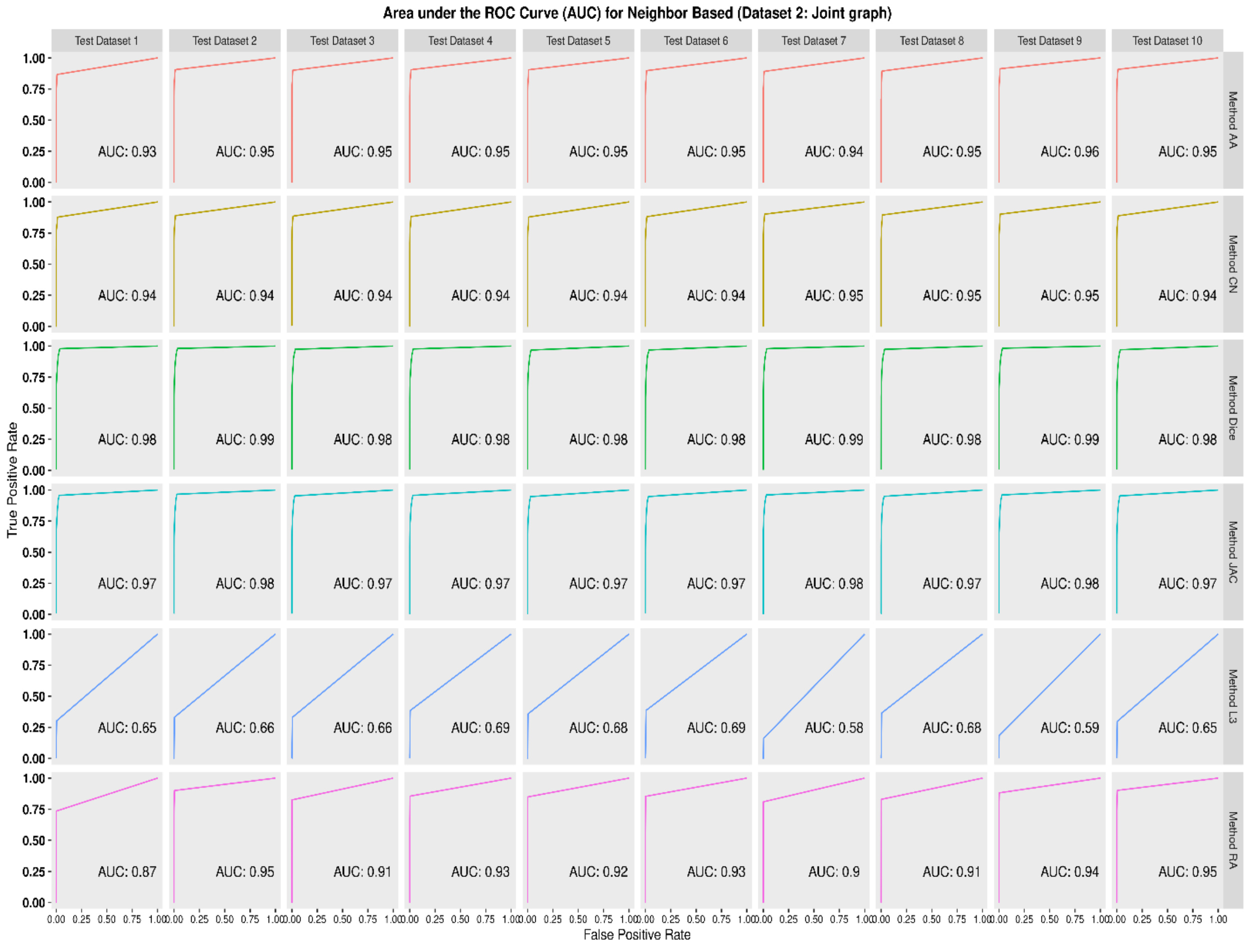

Figure S12: Area Under the Curve (AUC) for neighborhood-based similarity-based (dataset 2: joint graph). This figure was generated using ggplot2 library from R v3.6.3.

10. Precision-Recall Curve (PRC)

A precision-recall curve (also known as a PR Curve) is a graph that shows the precision (y-axis) and recall (x-axis) for various probability thresholds. We calculated precision and recall as follows:

$$Precision = \frac{True\ Positive}{True\ Positive + False\ Positive}$$

$$Recall = \frac{True\ Positive}{True\ Positive + False\ Negative}$$

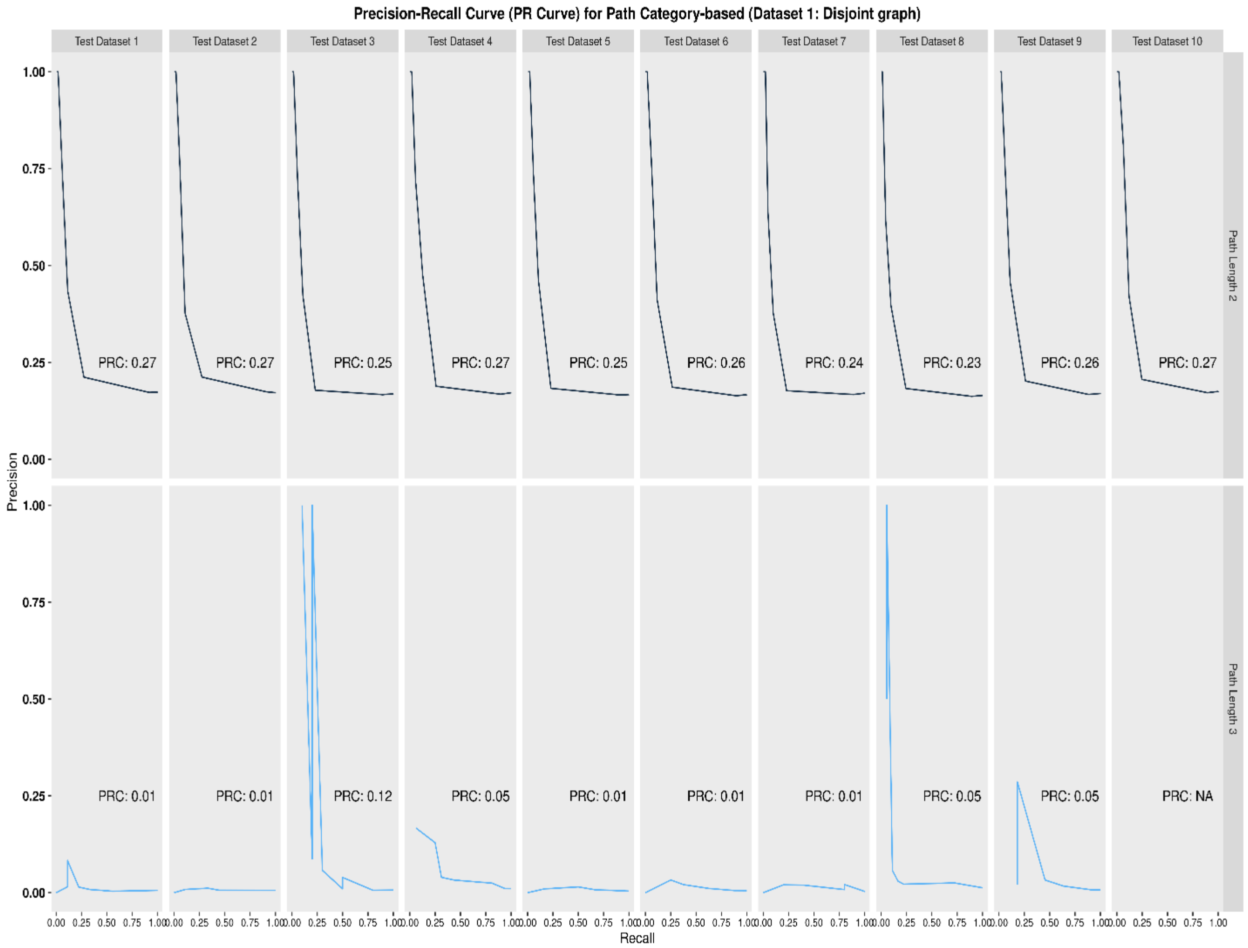

Figure S13: Precision-Recall Curve (PRC) for path category-based (dataset 1: disjoint graph). This figure was generated using ggplot2 library from R v3.6.3.

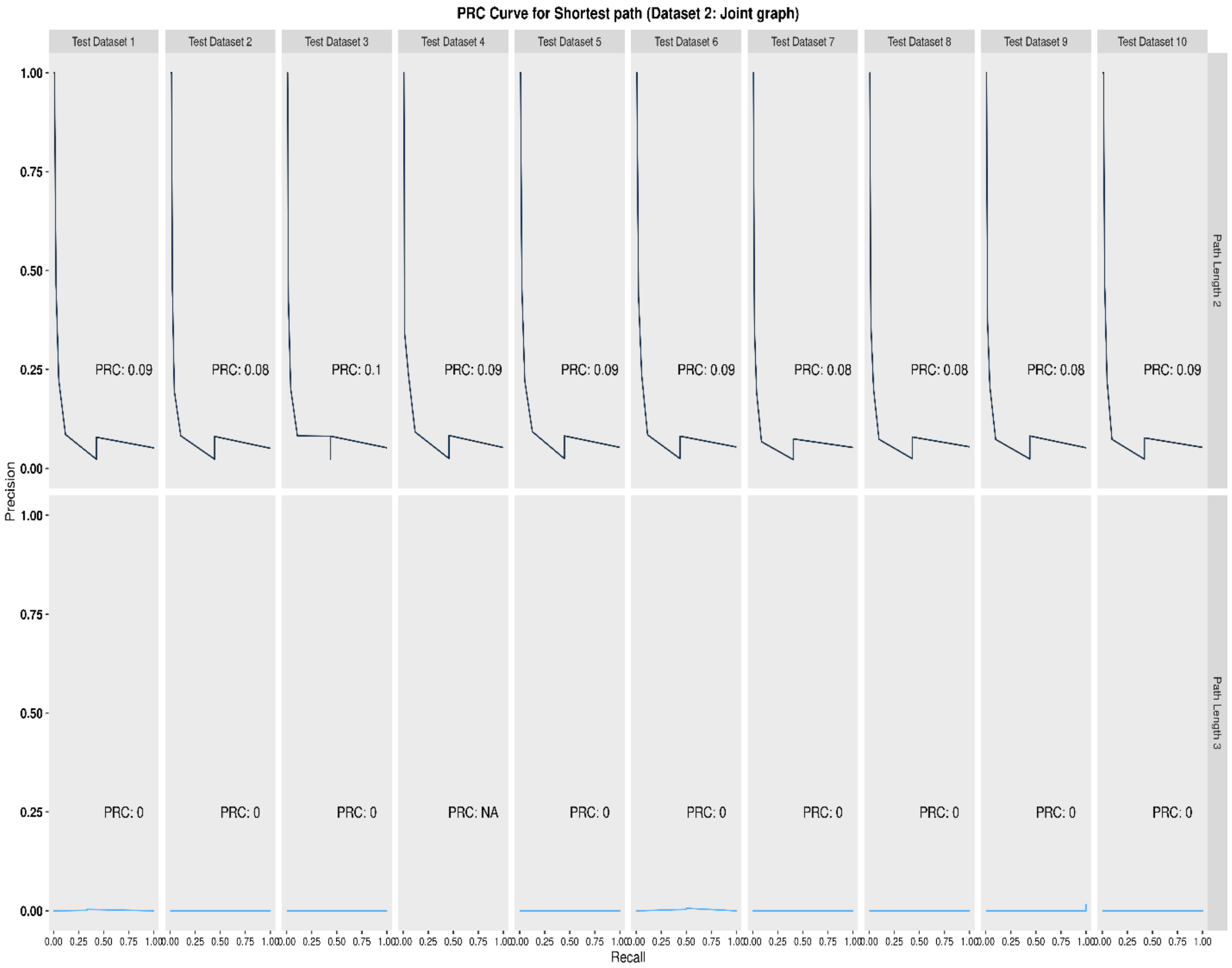

Figure S14: Precision-Recall Curve (PRC) for path category-based (dataset 2: joint graph). This figure was generated using ggplot2 library from R v3.6.3.

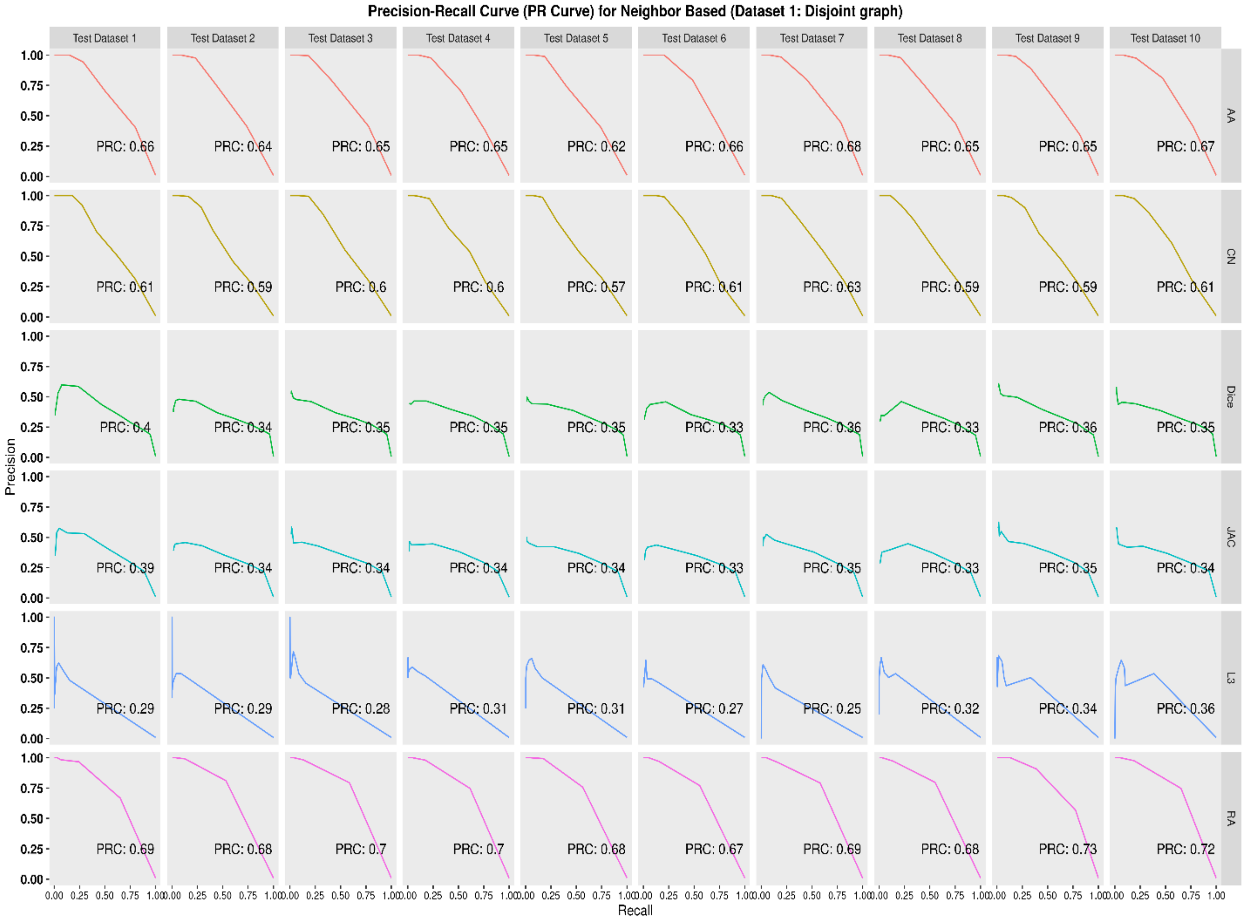

Figure S15: Precision-Recall Curve (PRC) for path neighborhood-based similarity-based (dataset 1: disjoint graph). This figure was generated using ggplot2 library from R v3.6.3.

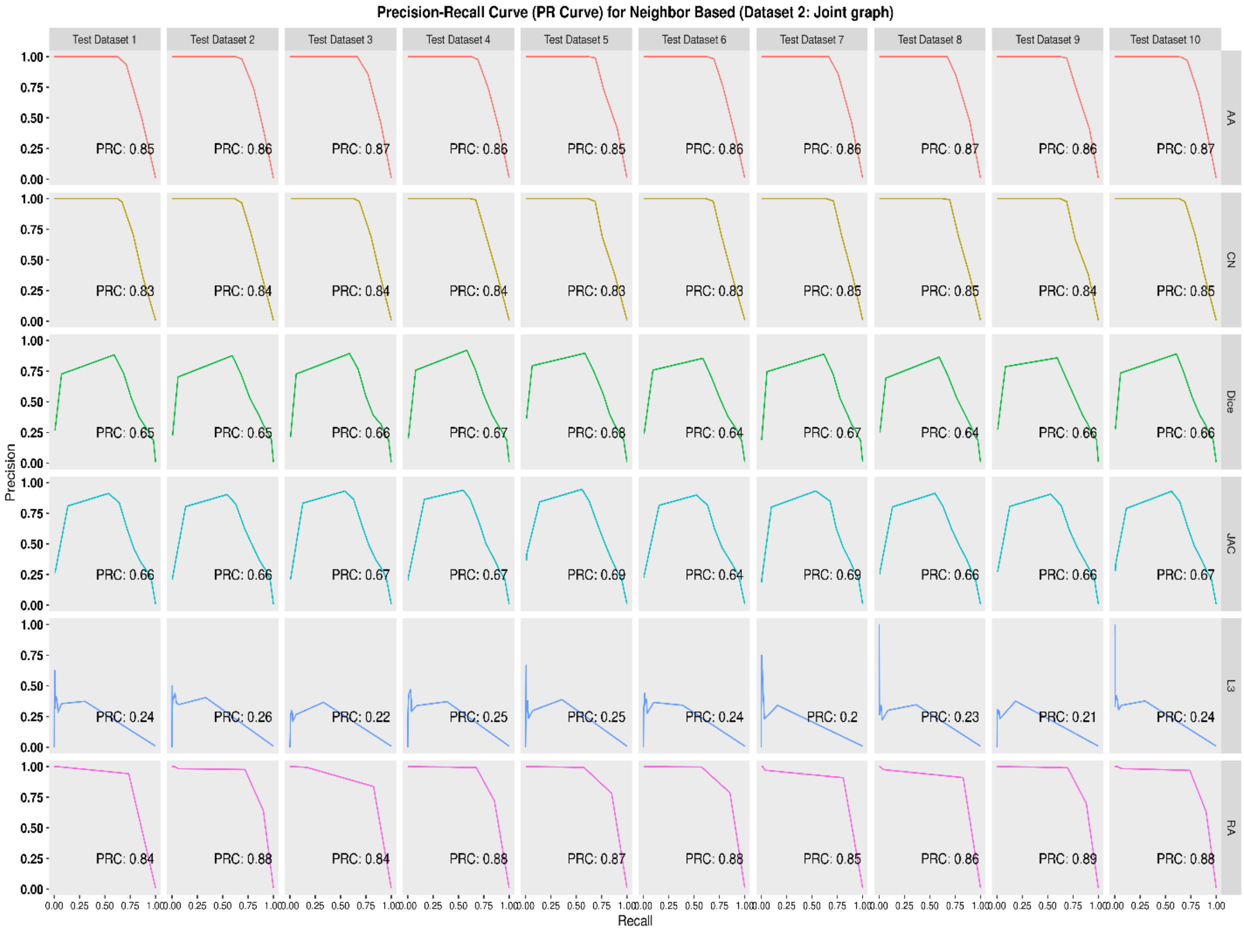

Figure S16: Precision-Recall Curve (PRC) for neighborhood-based similarity-based (dataset 2: joint graph). This figure was generated using ggplot2 library from R v3.6.3.

11. Updating Similarity Scores using Food-Compound Contribution

Table S2: Calculating New Score based on the Food Compound Contribution

nodeA	nodeB	Prior Score	Contribution (0~1)	New Score
DB00136	FOOD00165 _ FDB012362 _ beta-Sitosterol	0.6947674	0.3459079	0.2403255323224
FOOD00006 _ FDB000474 _ L-Lysine	FOOD00006 _ FDB000556 _ L-Alanine	0.6	0.009780473	0.005868284

12. Table S3 presents the number of links in the graph after applying different food compound contribution scores (based on the Tanimoto similarity threshold: 0.6).

Table S3: Number of links in the graph after applying different food compound contribution scores

Contribution Threshold	Total Links	DD Links	FF Links	FD Links
> 0.3	155,512	2,926	152,517	69
> 0.4	113,348	2,926	110,400	22
> 0.5	87,192	2,926	84,257	9
> 0.6	67,944	2,926	65,016	2

It should be noted that smaller numbers of FD links do not translate to the possible FDIs that can be examined. We have 87,192 and 92,143 (i.e., for disjoint and joint graphs respectively) possible FDIs when a threshold of > 0.5 is considered.

13.Results and Discussion

Table S4: Comparison of the precision@top (average), AUC, PRC over eight different methods on the disjoint graph network

**Method**	**'Precision@Top-1 (%)**	**Precision@Top-2 (%)**	**Precision@Top-5 (%)**	**AUC (%)**	**PRC (%)**
SP_2	**84 (±6.3)**	**60 (±5.3)**	**40 (±2.5)**	52 (±1.0)	26 (±1.0)
SP_3	05 (±5.6)	03 (±3.1)	02(±1.4)	59 (±23.0)	03 (±3.0)
AA	56 (±1.6)	36 (±1.0)	17 (±0.6)	88 (±.01)	65 (±1.7)
CN	53 (±1.5)	33 (±1.1)	17 (±0.4)	88 (±1.0)	60 (±1.6)
RA	64 (±1.7)	40 (±1.4)	17 (±0.6)	80 (±3.5)	**70 (±1.7)**
L3	58 (±1.9)	42 (±1.2)	17 (±0.6)	60 (±4)	30 (±3.1)
JAC	40 (±1.6)	31 (±0.5)	17 (±0.5)	94 (±0.4)	34 (±1.8)
Dice	40 (±1.6)	31 (±0.5)	17 (±0.5)	**97 (±0.7)**	35 (±2.0)

Table S5: Comparison of the precision@top (average), AUC, PRC over eight different methods on the joint graph network

**Method**	**Precision@Top-1 (%)**	**Precision@Top-2 (%)**	**Precision@Top-5 (%)**	**AUC (%)**	**PRC (%)**
SP 2	23 (±1.8)	15 (±1.5)	09 (±0.9)	38 (±1)	08 (±0.07)
SP 3	0.1 (±0.2)	0.1 (±0.1)	0.1 (±0.0)	88 (±31)	00 (±0)
AA	67 (±0.9)	37 (±0.5)	16 (±0.2)	95 (±0.6)	86 (±0.6)
CN	65 (±0.9	36 (±0.5)	16 (±0.2)	94 (±0.4)	84 (±0.7)

RA	**71 (±0.9)**	**39 (±0.5)**	**16 (±0.2)**	92 (±2.4)	**87 (±1.9)**
L3	31 (±1.2)	39 (±0.5)	16 (±0.2)	65 (±3.9)	23 (±2.0)
JAC	59 (±0.7)	35 (±0.4)	16 (±0.2)	97 (±0.3)	66 (±1.4)
Dice	59 (±0.7)	35 (±0.4)	16 (±0.2)	**98 (±0.2)**	65 (±1.4)

14. The Tanimoto coefficient score ranges for the disjoint and joint datasets are given below tables

Table S6: Disjoint Dataset DDIs Tanimoto Coefficient Score Ranges

**# DDI**	**> 0.95 & <=1**	**> 0.85 & <=0.95**	**> 0.75 & <= 0.85**	**> 0.65 & <=0.75**	**>0.55 & <=0.60**
2,926 (All)	77	143	317	1096	1293
1,706 (Matched with DrugBank)	60	100	215	626	705

Table S7: Joint Dataset DDIs Tanimoto Coefficient Score Ranges

**# DDI**	**> 0.95 & <=1**	**> 0.85 & <=0.95**	**> 0.75 & <= 0.85**	**> 0.65 & <=0.75**	**>0.55 & <=0.60**	**1e-5**
6,581 (All)	77	143	317	1096	1293	3655
4,178 (Matched with DrugBank)	60	100	215	626	705	2472

15. Details of the Gold Standard Dataset

The gold standard dataset was designed to act as a positive control to validate FDMine. A variety of the most notable interactions have been included to elucidate the ability of our model in confirming existing and known food-drug interactions. Although structural similarity may seem to be one of the most intuitive ways to investigate molecular food-drug interactions, there may be a wide variety of mechanisms at play when considering these types of interactions. To build the gold standard dataset, we have considered known food-drug interactions derived from 1) Cytochrome P450 based metabolic interactions 2) the effect of metallic ions such as K+ and Ca2+ on categories of drugs like ACE inhibitors and antibiotics respectively 3) the food-drug interactions derived from concomitant administration of opposing mechanisms/classes food-drug type interactions [ACE inhibitors vs beta antihypertensives] 4) Synergistic interactions, for example, monoamine oxidase inhibitors and tyramine. The approach we used to build this control dataset and capture a relatively diverse set of these kinds of interactions is discussed below. The gold standard dataset includes a total of 53 interactions across 26 unique foods and 14 categories of drugs.

The first part of this dataset consists of food-drug interactions that is prepared based on having a common target protein class CYP450. One of the common cases consist of the interactions as a result of the up-regulation or down-regulation of Cytochromes P450 (CYP450), a metabolic enzyme which plays a role in altering the pharmacodynamic profiles of about 90% of the currently available drugs [1]. The interactions of CYP450 related drugs include blood thinners, lipedemics, MAO inhibitors, beta blockers, antipyretics, ACE inhibitors and even antimetabolites. CYP450 refers to a group of enzymes including CYP1A2, CYP2C9, CYP2C19, CYP2D6, CYP3A4 and CYP3A5 [2]. Interactions can be in the shape of induction or inhibition of these enzymes. Since this class of enzymes are one of the many forms of defense mechanisms against xenobiotics or foreign chemical substances in the body, there can be serious implications for patient health. For example, one of the widely reported food interactions with CYP450 include grapefruits [3]. One of the constituents of grapefruit includes a class of compounds called furanocoumarins. Studies have shown that furanocoumarins affect mainly CYP3A4 [4]. Various kinds of furanocoumarins affect CYP450 in various ways [5]. For instance, dimeric furanocoumarins seem to affect CYP450 more than their monomeric analogues [6]. The inhibition of CYP450 can increase the bioavailability of several classes of drugs as mentioned above. For example, drugs containing moieties like furans, ter-amines and acetylene groups tend to get metabolized more by CYP450 [7]. Therefore, the inhibition of CYP450 by constituents like grapefruit tend to increase the bioavailability of drugs with such moieties. Therefore, it is of relevance to validate our model with these furanocoumarins with drugs targeting cytochromes (CYP3A4, CYP3A5, CYP450) like Simvastatin, Atorvastatin, Nifedipine, Buspirone and Fexofenadine among many others.

Another noteworthy group of food-drug interactions can be derived from vitamin K and Warfarin. vitamin K is a starting product that is hepatically used to produce various factors that help the blood clot during injury. Some of these factors include prothrombin, proconvertin, and Stuart-Prower factor. The blood clotting pathway is a very complex cascade of events that results in production of a fibrin mesh that effectively plugs the site of injury. It consists of an intrinsic and extrinsic pathway. The intrinsic pathway consists of the Hageman factor, Stuart-Prower factor, thromboplastin, Christmas factor, fibrinogen and prothrombin [8]. Excluding fibrin stabilizing factor, factor V and anti-hemophilic factor, all other blood clotting factors are zymogenic proteolytic enzymes that ultimately result in the activation of fibrin [8]. There are a total of 13 factors that participate in this biochemical cascade. Some of these clotting factors like Prothrombin are dependent on vitamin K and are derived from it [9]. The depletion of vitamin K reserves is mediated by Warfarin via VKORC1 [10] or vitamin K epoxide reductase complex 1, which deplete available vitamin K reserves thereby preventing the blood clotting factors required to form clots from functioning. This is the reason that eating cruciferous vegetables like Cabbage, Brussel sprouts, Kale, Turnips, and Broccoli can cause the effect of Warfarin to decrease as these foods are rich with vitamin K [11]. Warfarin inhibits vitamin K and causes the availability of vitamin K dependent factors to become unavailable [12]. Patients who suffer from blood clots in their arteries are usually prescribed warfarin, a commonly used blood thinner. Since warfarin quantitatively depletes the reserves of vitamin K, eating foods that contain substantially more vitamin K can in turn deplete the levels of Warfarin in the body. This can be a dangerous side effect for people taking Warfarin to prevent clots, for example patients that are to undergo angioplasty.

Another common food-drug interaction happens in foods rich in Tyramine, commonly found in fermented foods such as cheese, wine, deli meat, red wine, and tofu [13]. Tyramine is chemically hydroxy phenyl ethyl amine and closely resembles tyrosine and catecholamines like adrenaline and phenylephrine[14]. As a result, it causes sympatho-mimetic pressure responses or elevated blood pressure. Monoamine oxidase inhibitor (MAO) drugs like Isocarboxazid, Phenelzine, Selegiline, Tranylcypromine decrease [14] the catabolism or breakdown of Tyramine thereby leading to dangerous levels of hypertension. This can be

very deadly in patients with high blood pressure, who are also taking MAO inhibitors. This phenomenon is often referred to as the Tyramine pressor response.

One widely known food-drug interaction occurs as a result of the interaction of calcium with antibiotics. Foods rich in calcium such as milk and cheese tend to cause the chelation of antibiotics thereby rendering them unavailable for absorption. Tetracycline antibiotics like Doxycycline and Ciprofloxacin tend to chelate with calcium and are recommended to be not taken together as the chelated complex will have very poor bioavailability [15]. Although this is not a direct structural interaction, this is one of the many kinds of food drug interactions we wanted to test using our model. Another similar interaction based on metal ions includes the interaction of potassium with ACE inhibitors like Captopril, Enalapril and Lisinopril. ACE or Angiotensin converting enzyme is one of the prime components of a biochemical pathway called the Renin-Angiotensin system, which is responsible for the regulation of blood pressure that arises due to the amount of fluid in the body [16]. This ultimately leads to diuresis or egestion of fluid from the body in the form of urine. ACE is responsible for the conversion of Angiotensin I to Angiotensin II, which is a potent vasoconstrictor that mediates increase in blood pressure by inhibiting the reuptake of Norepinephrine, increases sodium retention while also helping the adrenal gland release more catecholamines, which are also potent vaso-constrictive agents [17]. This biochemical cascade also affects natriuresis via the release of Aldosterone and sodium retention, usually associated with high blood pressure [18]. In summary, ACE inhibitors directly inhibit ACE thereby preventing the above said cascade of biochemical reactions. Potassium, for instance, is found in bananas [19] and the ingestion with ACE inhibitors, also known as potassium sparing diuretics, represents a potentially dangerous interaction that can cause hyperkalemia induced effects such as irregular heartbeat.

Yet another widely known interaction is the interaction of liquorice and antihypertensives like ACE inhibitors and beta blockers. The hyptertensive effect of liquorice counteracts the hypotensive effects of the above mentioned drug categories via sodium retention and potassium excretion. The main constituent of liquorice that is responsible for these effects is glycyrrhizic acid. It undergoes transformation into glycyrrhetic acid, a compound with 200-1000 times the original potency of glycyrrhizic acid, by intestinal fauna, mediated by enzyme ß-glucuronidase. This glycyrrhetic acid is then absorbed into the liver and undergoes hepatic first pass metabolism into 3β-monoglucuronyl-18β-glycyrrhetinic acid via glucuronide [20].

Table S8: The gold standard evaluation of the disjoint dataset

Method	# FDI (as output from model training)	Matched FDI with Gold-standard	# Top_1	Matched Top_1	# Top_2	Matched Top_2	# Top_5	Matched Top_5
SP_L2	231	0	2	0	4	0	11	0
SP_L3	57	0	0	0	1	0	2	0
RA, AA, CN	138,994	0	1,389	0	2,779	0	6,949	0
L3	139,085	0	1,390	0	2,781	0	6,954	0

Table S9: The gold standard evaluation of the joint dataset

Method	# FDI (as output from model training)	Matched FDI with Gold-standard	# Top_1	Matched Top_1	# Top_2	Matched Top_2	# Top_5	Matched Top_5
SP_L2	15,199	5.77%	151	0	303	0	759	1.92%
SP_L3	172,798	**44.23%**	1,720	**13.46%**	3,455	**13.46%**	8,639	**26.92%**
RA, AA, CN	149,248	5.77%	1,492	0	2,984	3.85%	7,462	3.85%
L3	275,118	**46.15%**	2,749	3.85%	5,502	3.85%	13,755	5.77%

Table S10: The gold standard dataset

Food ID	Food ingredient	Drug	Reference
FOOD00887	vitamin K1	Warfarin	https://uihc.org/health-topics/warfarin-your-diet-and-vitamin-k-foods
FOOD00032	vitamin K1	Warfarin	
FOOD00390	vitamin K1	Warfarin	
FOOD00178	vitamin K1	Warfarin	
FOOD00027	vitamin K1	Warfarin	
FOOD00237	vitamin K1	Warfarin	
FOOD00036	vitamin K1	Warfarin	
FOOD00131	vitamin K1	Warfarin	
FOOD00034	vitamin K1	Warfarin	
FOOD00095	vitamin K1	Warfarin	
FOOD00888	vitamin K1	Warfarin	
-	vitamin K1	Warfarin	
FOOD00021	vitamin K1	Warfarin	
FOOD00241	vitamin K1	Warfarin	
FOOD00130	vitamin K1	Warfarin	
FOOD00256	furanocoumarin	Atenolol	https://www.fda.gov/consumers/consumer-updates/grapefruit-juice-and-some-drugs-dont-mix
FOOD00256	furanocoumarin	-	
FOOD00256	furanocoumarin	simvastatin	
FOOD00256	furanocoumarin	Atorvastatin	
FOOD00256	furanocoumarin	amlodipine	
FOOD00256	furanocoumarin	felodipine	
FOOD00256	furanocoumarin	isradipine	
FOOD00256	furanocoumarin	lacidipine	
FOOD00256	furanocoumarin	lercanidipine	
FOOD00256	furanocoumarin	nicardipine	

FOOD00256	furanocoumarin	nifedipine	
FOOD00256	furanocoumarin	nimodipine	
FOOD00256	furanocoumarin	verapamil	
FOOD00256	furanocoumarin	Ciclosporin	
FOOD00612	Tyramine	Isocarboxazid	https://www.ncbi.nlm.nih.gov/books/NBK539848/
FOOD00612	Tyramine	Phenelzine	
FOOD00612	Tyramine	Selegiline	
FOOD00612	Tyramine	Tranylcypromine	
FOOD00618	Calcium	Doxycycline	https://pubmed.ncbi.nlm.nih.gov/2767766/
FOOD00617	Calcium	Ciprofloxacin	https://pubmed.ncbi.nlm.nih.gov/1934862/
FOOD00617	daidzin, daidzein	Propranolol	
FOOD00619	Albumin	Propranolol	
FOOD00696		Propranolol	https://pubmed.ncbi.nlm.nih.gov/2899510/
FOOD00627	glycyrrhizin	Acebutolol	https://www.sciencedirect.com/topics/pharmacology-toxicology-and-pharmaceutical-science/liquorice#:~:text=The%20hypertensive%20effect%20of%20licorice,to%20its%20potential%20harmful%20effects.
FOOD00627	glycyrrhizin	Atenolol	
FOOD00627	glycyrrhizin	Bisoprolol	
FOOD00627	glycyrrhizin	Metoprolol	
FOOD00627	glycyrrhizin	Nadolol	
FOOD00627	glycyrrhizin	Nebivolol	
FOOD00627	glycyrrhizin	Propranolol	
FOOD00056	Hesperidin	celiprolol	https://onlinelibrary.wiley.com/doi/abs/10.1002/bdd.603#:~:text=In%20conclusion%2C%20it%20has%20been,to%20some%20beta%2Dblocking%20drugs.
FOOD00105	Pectin	acetaminophen	https://www.sciencedirect.com/science/article/abs/pii/S0022354915381582
FOOD00245	Pectin	acetaminophen	
FOOD00056	Pectin	acetaminophen	
FOOD00618	Xanthine Oxidase	Mercaptopurine	https://pubmed.ncbi.nlm.nih.gov/18045784/
FOOD00208	Potassium	captopril	https://jurnal.unej.ac.id/index.php/JAMS/article/view/8672
FOOD00208	Potassium	enalapril	
FOOD00208	Potassium	lisinopril	

16. Batch-1 Description and Result

The Tanimoto coefficient is applied on the datasets we made form DrugBank and FooDB. A structure similarity profile is created for all drugs and food compounds. After a preliminary investigation and testing, we have set the threshold value of 0.6 for the Tanimoto coefficient similarity. Later we calculate the food

compound's contribution in a food. Then, we multiplied the similarity score by the contribution of the food compound's score. Here, we preserved the similarity scores for the drugs pair. After updating the similarity scores in the graph, we have considered a contribution score threshold of 0.5.

Table S1 refers the top FDIs (based on score) after applying the path category-based (path length 2) method over the disjoint graph. In Table S1, Records might appear repeated, but food item IDs are different in this table. Table S2 refers the top 20 FDIs (based on the unique drug and food compound) after applying path category-based (path length 2) method over disjoint and joint graphs. Table S3 refers to the top 10 FDIs (based on the unique food and food compound) found using the joint graph network and neighborhood-based similarity-based link prediction algorithms.

Table S11: Top 10 FDIs found from path category-based (path length-2) method over disjoint graph. Records might appear repeated, but food item IDs are different in this table.

Top#	Food Name Compound ID Compound Name	Drug ID (Name)	Score
1	FOOD00006 _ FDB012858 _ Oleic acid	DB01080 (Vigabatrin)	1.359791
2	FOOD00009 _ FDB012858 _ Oleic acid	DB01080 (Vigabatrin)	1.359791
3	FOOD00011 _ FDB012858 _ Oleic acid	DB01080 (Vigabatrin)	1.359791
4	FOOD00012 _ FDB012858 _ Oleic acid	DB01080 (Vigabatrin)	1.359791
5	FOOD00015 _ FDB012858 _ Oleic acid	DB01080 (Vigabatrin)	1.359791
6	FOOD00016 _ FDB012858 _ Oleic acid	DB01080 (Vigabatrin)	1.359791
7	FOOD00017 _ FDB012858 _ Oleic acid	DB01080 (Vigabatrin)	1.359791
8	FOOD00021 _ FDB012858 _ Oleic acid	DB01080 (Vigabatrin)	1.359791
9	FOOD00024 _ FDB012858 _ Oleic acid	DB01080 (Vigabatrin)	1.359791
10	FOOD00026 _ FDB012858 _ Oleic acid	DB01080 (Vigabatrin)	1.359791

Table S12: Top 20 FDIs from path category-based (path length-2) method over disjoint and joint graph

Top#	Food Name Compound ID Compound Name	Drug ID (Name)	Score
**1**	FOOD00006 _ FDB012858 _ Oleic acid	DB01080 (Vigabatrin)	1.35979125586455
**2**	FOOD00099 _ FDB004287 _ Erucic acid	DB01080 (Vigabatrin)	1.35979125586455
**3**	FOOD00151 _ FDB002951 _ Elaidic acid	DB01080 (Vigabatrin)	1.35979125586455

**4**	FOOD00006 _ FDB012858 _ Oleic acid	DB03756 (Doconexent)	1.34065345300424
**5**	FOOD00099 _ FDB004287 _ Erucic acid	DB03756 (Doconexent)	1.34065345300424
**6**	FOOD00151 _ FDB002951 _ Elaidic acid	DB03756 (Doconexent)	1.34065345300424
**7**	FOOD00006 _ FDB012858 _ Oleic acid	DB00230 (Pregabalin)	1.3372149004032
**8**	FOOD00099 _ FDB004287 _ Erucic acid	DB00230 (Pregabalin)	1.3372149004032
**9**	FOOD00151 _ FDB002951 _ Elaidic acid	DB00230 (Pregabalin)	1.3372149004032
**10**	FOOD00009 _ FDB012760 _ (Z,Z)-9,12-Octadecadienoic acid	DB01080 (Vigabatrin)	1.27297207949509
**11**	FOOD00009 _ FDB012462 _ alpha-Linolenic acid	DB01080 (Vigabatrin)	1.26202848583508
**12**	FOOD00062 _ FDB012858 _ Oleic acid	DB00996 (Gabapentin)	1.26095178679852
**13**	FOOD00009 _ FDB012760 _ (Z,Z)-9,12-Octadecadienoic acid	DB03756 (Doconexent)	1.25383427663478
**14**	FOOD00009 _ FDB012760 _ (Z,Z)-9,12-Octadecadienoic acid	DB00230 (Pregabalin)	1.25039572403374
**15**	FOOD00009 _ FDB012462 _ alpha-Linolenic acid	DB03756 (Doconexent)	1.24289068297476
**16**	FOOD00009 _ FDB012462 _ alpha-Linolenic acid	DB00230 (Pregabalin)	1.23945213037372
**17**	FOOD00006 _ FDB001131 _ Sucrose	DB00273 (Topiramate)	1.2391096865889
**18**	FOOD00850 _ FDB001353 _ Uttronin A	DB00273 (Topiramate)	1.22885798978636
**19**	FOOD00085 _ FDB000661 _ Raffinose	DB00273 (Topiramate)	1.22704766985254
**20**	FOOD00085 _ FDB005666 _ Verbascose	DB00273 (Topiramate)	1.22704766985254

Table S13: Top 10 FDIs from Neighborhood-based Similarity-based method over joint graph

Top#	Food Name Compound ID Compound Name	Drug ID (Name)	Method	Score
**1**	FOOD00062 _ FDB012858 _ Oleic acid	DB00996 (Gabapentin)	AA	0.910239200
**2**	FOOD00525 _ FDB012858 _ Oleic acid	DB00996 (Gabapentin)	AA	0.910239200
**3**	FOOD00006 _ FDB001131 _ Sucrose	DB00273 (Topiramate)	AA	0.545550800
**4**	FOOD00006 _ FDB011824 _ beta-D-Glucopyranose	DB00273 (Topiramate)	AA	0.545550800
**5**	FOOD00006 _ FDB012528 _ D-Fructose	DB00273 (Topiramate)	AA	0.545550800
**6**	FOOD00012 _ FDB001128 _ Starch	DB00273 (Topiramate)	AA	0.545550800
**7**	FOOD00012 _ FDB001131 _ Sucrose	DB00273 (Topiramate)	AA	0.545550800
**8**	FOOD00012 _ FDB001182 _ Cellulose	DB00273 (Topiramate)	AA	0.545550800
**9**	FOOD00012 _ FDB011824 _ beta-D-Glucopyranose	DB00273 (Topiramate)	AA	0.545550800
**10**	FOOD00012 _ FDB012528 _ D-Fructose	DB00273 (Topiramate)	AA	0.545550800
**1**	FOOD00006 _ FDB001131 _ Sucrose	DB00273 (Topiramate)	CN	3
**2**	FOOD00006 _ FDB011824 _ beta-D-Glucopyranose	DB00273 (Topiramate)	CN	3
**3**	FOOD00006 _ FDB012528 _ D-Fructose	DB00273 (Topiramate)	CN	3
**4**	FOOD00012 _ FDB001128 _ Starch	DB00273 (Topiramate)	CN	3
**5**	FOOD00012 _ FDB001131 _ Sucrose	DB00273 (Topiramate)	CN	3
**6**	FOOD00012 _ FDB001182 _ Cellulose	DB00273 (Topiramate)	CN	3

**7**	FOOD00012 _ FDB011824 _ beta-D-Glucopyranose	DB00273 (Topiramate)	CN	3
**8**	FOOD00012 _ FDB012528 _ D-Fructose	DB00273 (Topiramate)	CN	3
**9**	FOOD00016 _ FDB001182 _ Cellulose	DB00273 (Topiramate)	CN	3
**10**	FOOD00017 _ FDB001141 _ Inulin	DB00273 (Topiramate)	CN	3
**1**	FOOD00121 _ FDB012858 _ Oleic acid	DB01080 (Vigabatrin)	L3	0.012168580
**2**	FOOD00042 _ FDB012760 _ (Z,Z)-9,12-Octadecadienoic acid	DB01080 (Vigabatrin)	L3	0.012168580
**3**	FOOD00326 _ FDB012858 _ Oleic acid	DB01080 (Vigabatrin)	L3	0.010614639
**4**	FOOD00283 _ FDB012760 _ (Z,Z)-9,12-Octadecadienoic acid	DB01080 (Vigabatrin)	L3	0.010614639
**5**	FOOD00777 _ FDB003010 _ Dodecanoic acid	DB01080 (Vigabatrin)	L3	0.005625870
**6**	FOOD00139 _ FDB001128 _ Starch	DB00273 (Topiramate)	L3	0.004414203
**7**	FOOD00139 _ FDB001128 _ Starch	DB00273 (Topiramate)	L3	0.004414203
**8**	FOOD00063 _ FDB001128 _ Starch	DB00273 (Topiramate)	L3	0.004383395
**9**	FOOD00323 _ FDB001128 _ Starch	DB00273 (Topiramate)	L3	0.004383395
**10**	FOOD00323 _ FDB001128 _ Starch	DB00273 (Topiramate)	L3	0.004383395
**1**	FOOD00062 _ FDB012858 _ Oleic acid	DB00996 (Gabapentin)	RA	0.333333333
**2**	FOOD00525 _ FDB012858 _ Oleic acid	DB00996 (Gabapentin)	RA	0.333333333
**3**	FOOD00006 _ FDB001131 _ Sucrose	DB00273 (Topiramate)	RA	0.012326489
**4**	FOOD00006 _ FDB011824 _ beta-D-Glucopyranose	DB00273 (Topiramate)	RA	0.012326489
**5**	FOOD00006 _ FDB012528 _ D-Fructose	DB00273 (Topiramate)	RA	0.012326489
**6**	FOOD00012 _ FDB001128 _ Starch	DB00273 (Topiramate)	RA	0.012326489
**7**	FOOD00012 _ FDB001131 _ Sucrose	DB00273 (Topiramate)	RA	0.012326489
**8**	FOOD00012 _ FDB001182 _ Cellulose	DB00273 (Topiramate)	RA	0.012326489
**9**	FOOD00012 _ FDB011824 _ beta-D-Glucopyranose	DB00273 (Topiramate)	RA	0.012326489
**10**	FOOD00012 _ FDB012528 _ D-Fructose	DB00273 (Topiramate)	RA	0.012326489

17. Batch-2 Description and Results

The Tanimoto coefficient is applied on the datasets we made form DrugBank and FooDB. A structure similarity profile is created for all drugs and food compounds. After a preliminary investigation and testing, we have set the threshold value of 0.6 for the Tanimoto coefficient similarity. Later we calculate the food compound's contribution in a food. Then, we multiplied the similarity score by the contribution of the food compound's score. Here, we preserved the similarity scores for the drugs pair. After updating the similarity scores in the graph, we have considered a second threshold of 0.3.

Table S1 refers the top 25 FDIs (based on the unique drug and food compound) using path category-based (path length 2) over the disjoint graph. Table S2 refers the top 25 FDIs (based on the unique drug and food compound) using path ategory-based (path length 2) over the joint graph. Table S3 refers to the common top 20 FDIs (based on the unique drug and food compound) using path category-based (path length 2) over the joint graph. Table S4 refers the top 25 FDIs (based on the unique food and food compound) using neighborhood-based similarity-based methods over the joint graph.

Table S14: Top 25 FDIs found from path category-based (path length-2) method over disjoint graph

SL	Drug	Food	Score
1	DB00195	FOOD00137 _ FDB012392 _ 1-Methoxy-4-(2-propenyl)benzene	1.31389823
2	DB00818	FOOD00089 _ FDB014512 _ 5-Isopropyl-2-methylphenol	1.29332913
3	DB00996	FOOD00062 _ FDB012858 _ Oleic acid	1.26095179
4	DB00335	FOOD00137 _ FDB012392 _ 1-Methoxy-4-(2-propenyl)benzene	1.25508236
5	DB00187	FOOD00137 _ FDB012392 _ 1-Methoxy-4-(2-propenyl)benzene	1.23189196
6	DB00818	FOOD00056 _ FDB014795 _ 2-Isopropyl-5-methylphenol	1.17521806
7	DB00368	FOOD00179 _ FDB012171 _ Eugenol	1.1414441
8	DB00612	FOOD00137 _ FDB012392 _ 1-Methoxy-4-(2-propenyl)benzene	1.13565637
9	DB06804	FOOD00019 _ FDB012392 _ 1-Methoxy-4-(2-propenyl)benzene	1.12878807
10	DB00668	FOOD00179 _ FDB012171 _ Eugenol	1.10561558
11	DB00264	FOOD00019 _ FDB012392 _ 1-Methoxy-4-(2-propenyl)benzene	1.09461935
12	DB00273	FOOD00290 _ FDB001128 _ Starch	1.07584505
13	DB00841	FOOD00179 _ FDB012171 _ Eugenol	1.06464978
14	DB01580	FOOD00137 _ FDB012392 _ 1-Methoxy-4-(2-propenyl)benzene	1.04581873
15	DB01359	FOOD00137 _ FDB012392 _ 1-Methoxy-4-(2-propenyl)benzene	1.03273112
16	DB00571	FOOD00137 _ FDB012392 _ 1-Methoxy-4-(2-propenyl)benzene	1.03058693
17	DB01182	FOOD00137 _ FDB012392 _ 1-Methoxy-4-(2-propenyl)benzene	1.02408808
18	DB00818	FOOD00068 _ FDB005326 _ alpha-Curcumene	1.02034449
19	DB06804	FOOD00012 _ FDB012373 _ Chavicol	1.01973566
20	DB00388	FOOD00179 _ FDB012171 _ Eugenol	1.0168991
21	DB00968	FOOD00179 _ FDB012171 _ Eugenol	0.99
22	DB00818	FOOD00067 _ FDB008492 _ Cuminyl alcohol	0.99
23	DB00264	FOOD00012 _ FDB012373 _ Chavicol	0.99
24	DB00818	FOOD00013 _ FDB017358 _ 1-Isopropyl-4-methylbenzene	0.98
25	DB00818	FOOD00051 _ FDB012268 _ Isopropylbenzene	0.98

Table S15: Top 25 FDIs found from path category-based (path length-2) method over joint graph

SL	Drug	Food	Score
1	DB00195	FOOD00137 _ FDB012392 _ 1-Methoxy-4-(2-propenyl)benzene	1.31389823
2	DB00818	FOOD00089 _ FDB014512 _ 5-Isopropyl-2-methylphenol	1.29332913
3	DB00996	FOOD00062 _ FDB012858 _ Oleic acid	1.26095179
4	DB00335	FOOD00137 _ FDB012392 _ 1-Methoxy-4-(2-propenyl)benzene	1.25508236
5	DB00187	FOOD00137 _ FDB012392 _ 1-Methoxy-4-(2-propenyl)benzene	1.23189196
6	DB00818	FOOD00056 _ FDB014795 _ 2-Isopropyl-5-methylphenol	1.17521806

7	DB00368	FOOD00179 _ FDB012171 _ Eugenol	1.1414441
8	DB00612	FOOD00137 _ FDB012392 _ 1-Methoxy-4-(2-propenyl)benzene	1.13565637
9	DB06804	FOOD00019 _ FDB012392 _ 1-Methoxy-4-(2-propenyl)benzene	1.12878807
10	DB00668	FOOD00179 _ FDB012171 _ Eugenol	1.10561558
11	DB00264	FOOD00019 _ FDB012392 _ 1-Methoxy-4-(2-propenyl)benzene	1.09461935
12	DB00273	FOOD00290 _ FDB001128 _ Starch	1.07584505
13	DB00841	FOOD00179 _ FDB012171 _ Eugenol	1.06464978
14	DB01580	FOOD00137 _ FDB012392 _ 1-Methoxy-4-(2-propenyl)benzene	1.04581873
15	DB01359	FOOD00137 _ FDB012392 _ 1-Methoxy-4-(2-propenyl)benzene	1.03273112
16	DB00571	FOOD00137 _ FDB012392 _ 1-Methoxy-4-(2-propenyl)benzene	1.03058693
17	DB01182	FOOD00137 _ FDB012392 _ 1-Methoxy-4-(2-propenyl)benzene	1.02408808
18	DB00818	FOOD00068 _ FDB005326 _ alpha-Curcumene	1.02034449
19	DB06804	FOOD00012 _ FDB012373 _ Chavicol	1.01973566
20	DB00388	FOOD00179 _ FDB012171 _ Eugenol	1.0168991
21	DB00199	FOOD00009 _ FDB002677 _ Hesperetin	1.00001
22	DB00208	FOOD00009 _ FDB002677 _ Hesperetin	1.00001
23	DB00215	FOOD00009 _ FDB002677 _ Hesperetin	1.00001
24	DB00244	FOOD00009 _ FDB002677 _ Hesperetin	1.00001
25	DB00283	FOOD00009 _ FDB002677 _ Hesperetin	1.00001

Table S16: Top 20 common FDIs found from path category-based (path length-2) method over disjoint and joint graph

SL	Drug	Food	Score
1	DB00195	FOOD00137 _ FDB012392 _ 1-Methoxy-4-(2-propenyl)benzene	1.31389823
2	DB00818	FOOD00089 _ FDB014512 _ 5-Isopropyl-2-methylphenol	1.29332913
3	DB00996	FOOD00062 _ FDB012858 _ Oleic acid	1.26095179
4	DB00335	FOOD00137 _ FDB012392 _ 1-Methoxy-4-(2-propenyl)benzene	1.25508236
5	DB00187	FOOD00137 _ FDB012392 _ 1-Methoxy-4-(2-propenyl)benzene	1.23189196
6	DB00818	FOOD00056 _ FDB014795 _ 2-Isopropyl-5-methylphenol	1.17521806
7	DB00368	FOOD00179 _ FDB012171 _ Eugenol	1.1414441
8	DB00612	FOOD00137 _ FDB012392 _ 1-Methoxy-4-(2-propenyl)benzene	1.13565637
9	DB06804	FOOD00019 _ FDB012392 _ 1-Methoxy-4-(2-propenyl)benzene	1.12878807
10	DB00668	FOOD00179 _ FDB012171 _ Eugenol	1.10561558
11	DB00264	FOOD00019 _ FDB012392 _ 1-Methoxy-4-(2-propenyl)benzene	1.09461935
12	DB00273	FOOD00290 _ FDB001128 _ Starch	1.07584505
13	DB00841	FOOD00179 _ FDB012171 _ Eugenol	1.06464978
14	DB01580	FOOD00137 _ FDB012392 _ 1-Methoxy-4-(2-propenyl)benzene	1.04581873
15	DB01359	FOOD00137 _ FDB012392 _ 1-Methoxy-4-(2-propenyl)benzene	1.03273112
16	DB00571	FOOD00137 _ FDB012392 _ 1-Methoxy-4-(2-propenyl)benzene	1.03058693
17	DB01182	FOOD00137 _ FDB012392 _ 1-Methoxy-4-(2-propenyl)benzene	1.02408808
18	DB00818	FOOD00068 _ FDB005326 _ alpha-Curcumene	1.02034449
19	DB06804	FOOD00012 _ FDB012373 _ Chavicol	1.01973566
20	DB00388	FOOD00179 _ FDB012171 _ Eugenol	1.0168991

Table S17: Top 25 FDIs from Neighborhood-based similarity-based methods method over the joint graph

SL	Drug	Food	Score	Method
1	DB00206	FOOD00186 _ FDB009721 _ Lignin	5.1008598423	AA
2	DB03756	FOOD00006 _ FDB012858 _ Oleic acid	2.7940418188	AA
3	DB03756	FOOD00009 _ FDB012462 _ alpha-Linolenic acid	2.7940418188	AA
4	DB03756	FOOD00009 _ FDB012760 _ (Z,Z)-9,12-Octadecadienoic acid	2.7940418188	AA
5	DB03756	FOOD00009 _ FDB012858 _ Oleic acid	2.7940418188	AA
6	DB03756	FOOD00011 _ FDB012462 _ alpha-Linolenic acid	2.7940418188	AA
7	DB03756	FOOD00011 _ FDB012760 _ (Z,Z)-9,12-Octadecadienoic acid	2.7940418188	AA
8	DB03756	FOOD00011 _ FDB012858 _ Oleic acid	2.7940418188	AA
9	DB03756	FOOD00012 _ FDB012462 _ alpha-Linolenic acid	2.7940418188	AA
10	DB03756	FOOD00012 _ FDB012760 _ (Z,Z)-9,12-Octadecadienoic acid	2.7940418188	AA
11	DB03756	FOOD00012 _ FDB012858 _ Oleic acid	2.7940418188	AA
12	DB03756	FOOD00013 _ FDB012462 _ alpha-Linolenic acid	2.7940418188	AA
13	DB03756	FOOD00013 _ FDB012760 _ (Z,Z)-9,12-Octadecadienoic acid	2.7940418188	AA
14	DB03756	FOOD00015 _ FDB012462 _ alpha-Linolenic acid	2.7940418188	AA
15	DB03756	FOOD00015 _ FDB012760 _ (Z,Z)-9,12-Octadecadienoic acid	2.7940418188	AA
16	DB03756	FOOD00015 _ FDB012858 _ Oleic acid	2.7940418188	AA
17	DB03756	FOOD00016 _ FDB012462 _ alpha-Linolenic acid	2.7940418188	AA
18	DB03756	FOOD00016 _ FDB012760 _ (Z,Z)-9,12-Octadecadienoic acid	2.7940418188	AA
19	DB03756	FOOD00016 _ FDB012858 _ Oleic acid	2.7940418188	AA
20	DB03756	FOOD00017 _ FDB012462 _ alpha-Linolenic acid	2.7940418188	AA
21	DB03756	FOOD00017 _ FDB012760 _ (Z,Z)-9,12-Octadecadienoic acid	2.7940418188	AA

22	DB03756	FOOD00017 _ FDB012858 _ Oleic acid	2.7940418188	AA
23	DB03756	FOOD00021 _ FDB012462 _ alpha-Linolenic acid	2.7940418188	AA
24	DB03756	FOOD00021 _ FDB012760 _ (Z,Z)-9,12-Octadecadienoic acid	2.7940418188	AA
25	DB03756	FOOD00021 _ FDB012858 _ Oleic acid	2.7940418188	AA
1	DB00206	FOOD00186 _ FDB009721 _ Lignin	22	CN
2	DB03756	FOOD00006 _ FDB012858 _ Oleic acid	17	CN
3	DB03756	FOOD00009 _ FDB012462 _ alpha-Linolenic acid	17	CN
4	DB03756	FOOD00009 _ FDB012760 _ (Z,Z)-9,12-Octadecadienoic acid	17	CN
5	DB03756	FOOD00009 _ FDB012858 _ Oleic acid	17	CN
6	DB03756	FOOD00011 _ FDB012462 _ alpha-Linolenic acid	17	CN
7	DB03756	FOOD00011 _ FDB012760 _ (Z,Z)-9,12-Octadecadienoic acid	17	CN
8	DB03756	FOOD00011 _ FDB012858 _ Oleic acid	17	CN
9	DB03756	FOOD00012 _ FDB012462 _ alpha-Linolenic acid	17	CN
10	DB03756	FOOD00012 _ FDB012760 _ (Z,Z)-9,12-Octadecadienoic acid	17	CN
11	DB03756	FOOD00012 _ FDB012858 _ Oleic acid	17	CN
12	DB03756	FOOD00013 _ FDB012462 _ alpha-Linolenic acid	17	CN
13	DB03756	FOOD00013 _ FDB012760 _ (Z,Z)-9,12-Octadecadienoic acid	17	CN
14	DB03756	FOOD00015 _ FDB012462 _ alpha-Linolenic acid	17	CN
15	DB03756	FOOD00015 _ FDB012760 _ (Z,Z)-9,12-Octadecadienoic acid	17	CN
16	DB03756	FOOD00015 _ FDB012858 _ Oleic acid	17	CN
17	DB03756	FOOD00016 _ FDB012462 _ alpha-Linolenic acid	17	CN
18	DB03756	FOOD00016 _ FDB012760 _ (Z,Z)-9,12-Octadecadienoic acid	17	CN
19	DB03756	FOOD00016 _ FDB012858 _ Oleic acid	17	CN

20	DB03756	FOOD00017 _ FDB012462 _ alpha-Linolenic acid	17	CN
21	DB03756	FOOD00017 _ FDB012760 _ (Z,Z)-9,12-Octadecadienoic acid	17	CN
22	DB03756	FOOD00017 _ FDB012858 _ Oleic acid	17	CN
23	DB03756	FOOD00021 _ FDB012462 _ alpha-Linolenic acid	17	CN
24	DB03756	FOOD00021 _ FDB012760 _ (Z,Z)-9,12-Octadecadienoic acid	17	CN
25	DB03756	FOOD00021 _ FDB012858 _ Oleic acid	17	CN
1	DB06804	FOOD00129 _ FDB014654 _ Myristicin	0.0154923936	L3
2	DB06804	FOOD00179 _ FDB012171 _ Eugenol	0.0138948964	L3
3	DB06804	FOOD00287 _ FDB012171 _ Eugenol	0.0136117656	L3
4	DB06804	FOOD00090 _ FDB002793 _ Anethole	0.0134151955	L3
5	DB06595	FOOD00186 _ FDB009721 _ Lignin	0.0080457224	L3
6	DB03756	FOOD00130 _ FDB012858 _ Oleic acid	0.0056661333	L3
7	DB03756	FOOD00140 _ FDB012858 _ Oleic acid	0.0056661333	L3
8	DB03756	FOOD00011 _ FDB012858 _ Oleic acid	0.0056126899	L3
9	DB03756	FOOD00148 _ FDB012858 _ Oleic acid	0.0056126899	L3
10	DB03756	FOOD00283 _ FDB012858 _ Oleic acid	0.0056126899	L3
11	DB13879	FOOD00336 _ FDB009721 _ Lignin	0.0055611791	L3
12	DB00988	FOOD00137 _ FDB012392 _ 1-Methoxy-4-(2-propenyl)benzene	0.0053589198	L3
13	DB00388	FOOD00197 _ FDB000567 _ L-DOPA	0.0052822140	L3
14	DB03756	FOOD00026 _ FDB012760 _ (Z,Z)-9,12-Octadecadienoic acid	0.0048688671	L3
15	DB03756	FOOD00316 _ FDB012760 _ (Z,Z)-9,12-Octadecadienoic acid	0.0048688671	L3
16	DB03756	FOOD00341 _ FDB012760 _ (Z,Z)-9,12-Octadecadienoic acid	0.0048688671	L3
17	DB03756	FOOD00052 _ FDB012760 _ (Z,Z)-9,12-Octadecadienoic acid	0.0048559821	L3

18	DB03756	FOOD00065 _ FDB012462 _ alpha-Linolenic acid	0.0048559821	L3
19	DB03756	FOOD00024 _ FDB012858 _ Oleic acid	0.0048445006	L3
20	DB03756	FOOD00052 _ FDB012858 _ Oleic acid	0.0048445006	L3
21	DB03756	FOOD00061 _ FDB012858 _ Oleic acid	0.0048445006	L3
22	DB03756	FOOD00130 _ FDB012760 _ (Z,Z)-9,12-Octadecadienoic acid	0.0048445006	L3
23	DB03756	FOOD00433 _ FDB012858 _ Oleic acid	0.0047983642	L3
24	DB03756	FOOD00777 _ FDB003010 _ Dodecanoic acid	0.0047543281	L3
25	DB01080	FOOD00042 _ FDB012760 _ (Z,Z)-9,12-Octadecadienoic acid	0.0039317135	L3
1	DB00206	FOOD00186 _ FDB009721 _ Lignin	0.297311868837	RA
2	DB00368	FOOD00197 _ FDB000567 _ L-DOPA	0.25	RA
3	DB00368	FOOD00179 _ FDB012171 _ Eugenol	0.166666666666	RA
4	DB00996	FOOD00062 _ FDB012858 _ Oleic acid	0.166666666666	RA
5	DB00996	FOOD00121 _ FDB012858 _ Oleic acid	0.166666666666	RA
6	DB00996	FOOD00182 _ FDB012858 _ Oleic acid	0.166666666666	RA
7	DB00996	FOOD00326 _ FDB012858 _ Oleic acid	0.166666666666	RA
8	DB00996	FOOD00525 _ FDB012858 _ Oleic acid	0.166666666666	RA
9	DB00187	FOOD00137 _ FDB012392 _ 1-Methoxy-4-(2-propenyl)benzene	0.111111111111	RA
10	DB00273	FOOD00004 _ FDB021453 _ Pectic acid	0.041061856930	RA
11	DB00273	FOOD00006 _ FDB001131 _ Sucrose	0.041061856930	RA
12	DB00273	FOOD00006 _ FDB011824 _ beta-D-Glucopyranose	0.041061856930	RA
13	DB00273	FOOD00006 _ FDB012528 _ D-Fructose	0.041061856930	RA
14	DB00273	FOOD00012 _ FDB001128 _ Starch	0.041061856930	RA
15	DB00273	FOOD00012 _ FDB001182 _ Cellulose	0.041061856930	RA
16	DB00273	FOOD00012 _ FDB011824 _ beta-D-Glucopyranose	0.041061856930	RA
17	DB00273	FOOD00012 _ FDB012528 _ D-Fructose	0.041061856930	RA
18	DB00273	FOOD00012 _ FDB021453 _ Pectic acid	0.041061856930	RA

19	DB00273	FOOD00015 _ FDB021453 _ Pectic acid	0.041061856930	RA
20	DB00273	FOOD00016 _ FDB001182 _ Cellulose	0.041061856930	RA
21	DB00273	FOOD00019 _ FDB001193 _ Maltose	0.041061856930	RA
22	DB00273	FOOD00021 _ FDB001131 _ Sucrose	0.041061856930	RA
23	DB00273	FOOD00022 _ FDB001128 _ Starch	0.041061856930	RA
24	DB00273	FOOD00022 _ FDB001182 _ Cellulose	0.041061856930	RA
25	DB00273	FOOD00038 _ FDB001128 _ Starch	0.041061856930	RA

18. Related Work

The following table contains a summary of related work

Table S18: Summary of related work

Author	Name of work	Year	Prediction	Approach
Reker, D. et al	Machine learning uncovers food- and excipient-drug interactions	2020	Food and excipient-drug interactions	Machine learning
Allahgholi, M., Rahmani, H., Javdani, D., Weiss, G. & Módos, D	ADDI: Recommending alternatives for drug-drug interactions with negative health effects	2020	drug-drug interactions with negative health effects	Deep learning
Feng, Y.-H., Zhang, S.-W. & Shi, J.-Y	DPDDI: a deep predictor for drug-drug interactions	2020	Drug-drug interactions	Deep learning
Lee, G., Park, C. & Ahn	Novel deep learning model for more accurate prediction of drug-drug interaction effects	2019	Drug-drug interaction	Deep learning
You, J., McLeod, R. D. & Hu, P.	Predicting drug-target interaction network using deep learning model	2019	Drug-target interactions	Deep learning
Ryu, J. Y., Kim, H. U. & Lee, S. Y	Deep learning improves prediction of drug-drug and drug-food interactions	2018	Drug-drug and Drug-food interaction	Deep learning

Olayan, R. S., Ashoor, H. & Bajic, V. B	DDR: efficient computational method to predict drug-target interactions using graph mining and machine learning approaches	2018	Drug-target interactions	Graph mining
Lu, Y., Guo, Y. & Korhonen, A	Link prediction in drug-target interactions network using similarity indices	2017	Drug-target interactions	Network topology
Ba-Alawi, W., Soufan, O., Essack, M., Kalnis, P. & Bajic, V. B	DASPfind: new efficient method to predict drug-target interactions	2016	Drug-target interactions	Graph mining
Fokoue, A., Sadoghi, M., Hassanzadeh, O. & Zhang, P	Predicting drug-drug interactions through large-scale similarity-based link prediction.	2016	Drug-drug interactions	Similarity-based link prediction.

19. Performance of the joint graph using different random seeds

To examine the performance of the joint graph with different random seeds, we have repeated our experiments using the joint dataset with path length of L=2, L=3, and neighborhood algorithms. In each run, we choose different random nodes to build the connections across the disjoint parts. The process is repeated ten times with different random seeds. The following table illustrates the results of precision@k for each seed.

Table S19: Comparison of the precision@top (average), over ten different seeds on the joint graph network (SP_2)

	Random seed 1 (FDMine results)	Random seed 2	Random seed 3	Random seed 4	Random seed 5	Random seed 6	Random seed 7	Random seed 8	Random seed 9	Random seed 10
**Precision@Top_1%**	23 (1.8)	19.50 (1.70)	21.44 (2.68)	21.94 (3.84)	20.55 (3.80)	23.07 (3.15)	19.24 (1.86)	20.18 (2.55)	20.22 (2.25)	25.53 (2.87)
**Precision@Top_2%**	15 (1.5)	12.92 (0.90)	14.14 (1.77)	14.53 (2.55)	14.03 (1.98)	15.82 (2.07)	13.03 (1.27)	13.46 (1.76)	13.89 (1.65)	16.93 (1.50)
**Precision@Top_5%**	09 (0.9)	8.41 (0.61)	9.10 (0.79)	9.21 (0.65)	8.68 (0.87)	10.01 (0.60)	8.50 (0.60)	8.83 (0.91)	8.83 (0.71)	10.22 (0.79)

Table S20: Comparison of the precision@top (average), over ten different seeds on the joint graph network (SP_3)

	Random seed 1 (FDMine results)	Random seed 2	Random seed 3	Random seed 4	Random seed 5	Random seed 6	Random seed 7	Random seed 8	Random seed 9	Random seed 10
**Precision@Top_1%**	0.10 (0.2)	0.24 (0.41)	0.12 (0.19)	0.17 (0.20)	0.23 (0.19)	0.45 (0.40)	0.11 (0.17)	0.19 (0.26)	0.35 (0.43)	0.13 (0.26)
**Precision@Top_2%**	0.10 (0.1)	0.14 (0.20)	0.22 (0.17)	0.16 (0.16)	0.19 (0.12)	0.32 (0.28)	0.09 (0.09)	0.22 (0.27)	0.25 (0.17)	0.14 (0.17)
**Precision@Top_5%**	0.10 (0.0)	0.12 (0.10)	0.11 (0.06)	0.11 (0.11)	0.11 (0.04)	0.18 (0.16)	0.07 (0.06)	0.11 (0.12)	0.14 (0.05)	0.11 (0.07)

Table S21: Comparison of the precision@top (average), over ten different seeds on the neighborhood algorithms

	Method	Random seed 1 (FDMine results)	Random seed 2	Random seed 3	Random seed 4	Random seed 5	Random seed 6	Random seed 7	Random seed 8	Random seed 9	Random seed 10
**Precision@Top_1%**	**AA**	67 (±0.9)	67.11 (1.06)	66.12 (0.86)	66.13 (0.91)	66.20 (1.40)	66.23 (0.89)	66.17 (0.65)	65.84 (1.60)	66.50 (1.18)	66.01 (0.89)
**Precision@Top_2%**		37 (±0.5)	37.31 (0.61)	36.64 (0.32)	36.72 (0.52)	36.60 (0.64)	36.85 (0.52)	36.92 (0.21)	36.76 (0.45)	36.90 (0.66)	36.92 (0.36)

**Precision@Top_5%**		16 (±0.2)	16.02 (0.22)	15.85 (0.13)	15.89 (0.11)	15.82 (0.19)	15.96 (0.12)	15.92 (0.10)	15.92 (0.11)	15.96 (0.25)	15.99 (0.08)
**Precision@Top_1%**	**CN**	65 (±0.9)	65.12 (0.99)	64.45 (1.26)	64.44 (1.12)	64.16 (35.68)	64.17 (0.82)	64.32 (0.59)	64.02 (1.70)	64.47 (1.14)	64.40 (0.87)
**Precision@Top_2%**		36 (±0.5)	36.34 (0.47)	35.63 (0.33)	35.81 (0.41)	35.68 (0.69)	35.85 (0.46)	35.83 (0.25)	35.71 (0.59)	35.82 (0.59)	35.98 (0.33)
**Precision@Top_5%**		16 (±0.2)	15.82 (0.23)	15.59 (0.17)	15.61 (0.15)	15.59 (0.21)	15.73 (0.14)	15.68 (0.11)	15.70 (0.13)	15.72 (0.24)	15.74 (0.07)
**Precision@Top_1%**	**RA**	71 (±0.9)	70.85 (1.21)	70.37 (0.56)	70.40 (0.80)	70.31 (0.89)	70.91 (0.66)	70.61 (0.45)	70.14 (1.04)	70.72 (1.34)	70.43 (0.73)
**Precision@Top_2%**		39 (±0.5)	38.64 (0.54)	38.35 (0.30)	38.35 (0.45)	38.08 (0.49)	38.52 (0.40)	38.56 (0.27)	38.33 (0.43)	38.47 (0.65)	38.61 (0.26)
**Precision@Top_5%**		16 (±0.2)	16.02 (0.22)	15.85 (0.13)	15.89 (0.11)	15.82 (0.19)	15.96 (0.12)	15.92 (0.10)	15.92 (0.11)	15.96 (0.25)	15.99 (0.08)
**Precision@Top_1%**	**L3**	31 (±1.2)	30.82 (1.17)	30.60 (0.79)	30.92 (1.02)	30.94 (0.69)	31.70 (1.13)	30.88 (1.19)	31.07 (1.33)	31.27 (0.74)	32.48 (1.23)
**Precision@Top_2%**		39 (±0.5)	38.96 (0.57)	38.64 (0.25)	38.47 (0.23)	38.25 (0.49)	39.24 (0.33)	38.65 (0.37)	38.52 (0.30)	38.57 (0.62)	39.26 (0.25)
**Precision@Top_5%**		16 (±0.2)	15.97 (0.22)	15.77 (0.11)	15.80 (0.11)	15.75 (0.18)	15.88 (0.13)	15.84 (0.09)	15.84 (0.11)	15.89 (0.24)	15.90 (0.07)
**Precision@Top_1%**	**JAC**	59 (±0.7)	58.82 (0.84)	58.61 (0.77)	58.22 (0.61)	57.52 (0.91)	59.01 (1.29)	58.17 (0.72)	57.87 (1.43)	58.12 (0.79)	58.92 (0.96)
**Precision@Top_2%**		35 (±0.4)	34.77 (0.50)	34.41 (0.35)	34.44 (0.31)	34.42 (0.44)	35.06 (0.29)	34.66 (0.29)	34.59 (0.50)	34.82 (0.58)	34.78 (0.33)
**Precision@Top_5%**		16 (±0.2)	15.97 (0.23)	15.81 (0.12)	15.85 (0.09)	15.77 (0.20)	15.90 (0.10)	15.89 (0.10)	15.89 (0.12)	15.94 (0.23)	15.93 (0.06)
**Precision@Top_1%**	**Dice**	59 (±0.7)	58.82 (0.84)	58.61 (0.77)	58.22 (0.61)	57.52 (0.91)	59.01 (1.29)	58.17 (0.72)	57.87 (1.43)	58.12 (0.79)	58.92 (0.96)

**Precision@Top_2%**		35 (±0.4)	34.77 (0.50)	34.41 (0.35)	34.44 (0.31)	34.42 (0.44)	35.06 (0.29)	34.66 (0.29)	34.59 (0.50)	34.82 (0.58)	34.78 (0.33)
**Precision@Top_5%**		16 (±0.2)	15.97 (0.23)	15.81 (0.12)	15.85 (0.09)	15.77 (0.20)	15.90 (0.10)	15.89 (0.10)	15.89 (0.12)	15.94 (0.23)	15.93 (0.06)

20. Different edge weight for the joint graph

The graph edge weight represents a similarity score. Yet, as the reviewers indicated, Eq. 3 of Dijkstra will select paths with minimum edge weights. Our purpose of adding an edge is to satisfy the requirement for algorithms such as Adamic and Adar Coefficient (AA), Common Neighbour (CN) (see lines 242-243 in the manuscript) for which it is sufficient to just add an edge without a weight. For a more general representation, we considered assigning a weight for the edge. We note that the choice of the weight can be subject to further research and analysis. In the current work, we considered an edge weight of 1e-5, which, in the case of Dijkstra's algorithm, will provide an increased probability of identifying connections across disjoint groups. As noted in our response #2, Dijkstra prefers these small weights and hence we achieve the aim of extending the disjoint graph to a joint version. We have considered an experiment with high edge weight (i.e., 1) to examine the effect on performance. For shortest path evaluation, the performance dropped when higher weights were assigned. The results are as follows:

Table S22: The results for the joint dataset (Random edge weight: 1) (Result is the avg of 10 times)

	FDMine (L_2; edge weight = 1e-5)	FDMine (L_2; edge weight = 1)	FDMine (L_3; edge weight = 1e-5)	FDMine (L_3; edge weight = 1)
**Precision@Top_1%**	0.23	0	0.01	0
**Precision@Top_2%**	0.15	0	0.01	0
**Precision@Top_5%**	0.09	0	0.01	0

Reference

[1] Gilani, B., & Cassagnol, M. (2021). Biochemistry, Cytochrome P450. In StatPearls. Treasure Island (FL): StatPearls Publishing

[2] de Andrés, F., Altamirano-Tinoco, C., Ramírez-Roa, R., Montes-Mondragón, C. F., Dorado, P., Peñas-Lledó, E. M., & LLerena, A. (2021). Relationships between CYP1A2, CYP2C9, CYP2C19, CYP2D6 and CYP3A4 metabolic phenotypes and genotypes in a Nicaraguan Mestizo population. The Pharmacogenomics Journal, 21(2), 140–151.

[3] Bailey, D. G., Dresser, G., & Arnold, J. M. O. (2013). Grapefruit-medication interactions: forbidden fruit or avoidable consequences? Journal de l'Association Medicale Canadienne [Canadian Medical Association Journal], 185(4), 309–316.

[4] Guo, L.-Q., & Yamazoe, Y. (2004). Inhibition of cytochrome P450 by furanocoumarins in grapefruit juice and herbal medicines. Acta Pharmacologica Sinica, 25(2), 129–136.

[5] Tassaneeyakul, W., Guo, L. Q., Fukuda, K., Ohta, T., & Yamazoe, Y. (2000). Inhibition selectivity of grapefruit juice components on human cytochromes P450. Archives of Biochemistry and Biophysics, 378(2), 356–363.

[6] Guo, L. Q., Taniguchi, M., Xiao, Y. Q., Baba, K., Ohta, T., & Yamazoe, Y. (2000). Inhibitory effect of natural furanocoumarins on human microsomal cytochrome P450 3A activity. The Japanese Journal of Pharmacology, 82(2), 122–129.

[7] Beck, T. C., Beck, K. R., Morningstar, J., Benjamin, M. M., & Norris, R. A. (2021). Descriptors of cytochrome inhibitors and useful machine learning based methods for the design of safer drugs. Pharmaceuticals (Basel, Switzerland), 14(5), 472.

[8] Chaudhry, R., Usama, S. M., & Babiker, H. M. (2020). Physiology, Coagulation Pathways. In StatPearls. Treasure Island (FL): StatPearls Publishing.

[9] Girolami, A., Ferrari, S., Cosi, E., Santarossa, C., & Randi, M. L. (2018). vitamin K-dependent coagulation factors that may be responsible for both bleeding and thrombosis (FII, FVII, and FIX). Clinical and Applied Thrombosis/Hemostasis, 24(9_suppl), 42S-47S.

[10] Patel, S., Singh, R., Preuss, C. V., & Patel, N. (2021). Warfarin. In StatPearls. Treasure Island (FL): StatPearls Publishing.

[11] Ovesen, L., Lyduch, S., & Idorn, M. L. (1988). The effect of a diet rich in brussels sprouts on warfarin pharmacokinetics. European Journal of Clinical Pharmacology, 34(5), 521–523.

[12] Wu, S., Chen, X., Jin, D.-Y., Stafford, D. W., Pedersen, L. G., & Tie, J.-K. (2018). Warfarin and vitamin K epoxide reductase: a molecular accounting for observed inhibition. Blood, 132(6), 647–657.

[13] Shulman, K. I., Walker, S. E., MacKenzie, S., & Knowles, S. (1989). Dietary restriction, tyramine, and the use of monoamine oxidase inhibitors. Journal of Clinical Psychopharmacology, 9(6), 397–402.

[14] Sub Laban, T., & Saadabadi, A. (2021). Monoamine Oxidase Inhibitors (MAOI). In StatPearls. Treasure Island (FL): StatPearls Publishing.

[15] Neuvonen, P. J. (1976). Interactions with the absorption of tetracyclines. Drugs, 11(1), 45–54.

[16] Fountain, J. H., & Lappin, S. L. (2021). Physiology, Renin Angiotensin System. In StatPearls. Treasure Island (FL): StatPearls Publishing.

[17] Herman, L. L., Padala, S. A., Ahmed, I., & Bashir, K. (2021). Angiotensin Converting Enzyme Inhibitors (ACEI). In StatPearls. Treasure Island (FL): StatPearls Publishing.

[18] Donckier, J. E., Kolanowski, J., Berbinschi, A., Gerard, G., & Ketelslegers, J. M. (1990). Relationship between natriuresis and changes in plasma atrial natriuretic factor, renin activity and aldosterone levels in fasting obese subjects. Diabete & Metabolisme, 16(4), 278–283.

[19] Olivia, Z., & Suryana, A. L. (2018). Effect of antihipertensive drugs and banana (Musa Sp.) to potassium serum levels of hypertensive wistar rats model. Journal of Agromedicine and Medical Sciences, 4(3), 121.

[20] Omar, H. R., Komarova, I., El-Ghonemi, M., Fathy, A., Rashad, R., Abdelmalak, H. D., … Camporesi, E. M. (2012). Licorice abuse: time to send a warning message. Therapeutic Advances in Endocrinology and Metabolism, 3(4), 125–138.
